# Are Autistic and Alexithymic Traits Distinct? A Factor-Analytic and Network Approach

**DOI:** 10.1007/s10803-021-05094-6

**Published:** 2021-06-01

**Authors:** Hélio Clemente Cuve, Jennifer Murphy, Hannah Hobson, Eri Ichijo, Caroline Catmur, Geoffrey Bird

**Affiliations:** 1 Department of Experimental Psychology, University of Oxford, Anna Watts Building, Radcliffe Observatory, Woodstock Rd, Oxford, OX2 6GG UK; 2 Department of Psychology, Royal Holloway, University of London, London, UK; 3 Department of Psychology, University of York, York, UK; 4 Department of Psychology, Institute of Psychiatry, Psychology and Neuroscience, King’s College London, London, UK; 5 Social, Genetic and Developmental Psychiatry Centre, Institute of Psychiatry, Psychology and Neuroscience, King’s College London, London, UK

**Keywords:** Autism, Alexithymia, Factor, Network, Separation

## Abstract

Despite the heterogeneity in autism, socioemotional difficulties are often framed as universal. Increasing evidence, however, suggests that socioemotional difficulties may be explained by alexithymia, a distinct yet frequently co-occurring condition. If, as some propose, autistic traits are responsible for socioemotional impairments, then alexithymia may itself be a symptom of autism. We aimed to determine whether alexithymia should be considered a product of autism or regarded as a separate condition. Using factor-analytic and network approaches, we provide evidence that alexithymic and autistic traits are distinct. We argue that: (1) models of socioemotional processing in autism should conceptualise difficulties as intrinsic to alexithymia; and (2) assessment of alexithymia is crucial for diagnosis and personalised interventions.

**Supplementary Information:**

The online version contains supplementary material available at 10.1007/s10803-021-05094-6.

## Introduction

Autism spectrum disorder (‘autism’) is a multi-dimensional condition defined by difficulties with social interaction and communication, and restricted and repetitive interests and behaviours (APA, 2013). It is well-recognised that autism is a highly heterogenous condition (Martinez-Murcia et al., 2017; Mottron & Bzdok, 2020), and this heterogeneity is particularly apparent in socioemotional functioning (emotion recognition and emotional reciprocity). Despite assertions that socioemotional difficulties are a ‘hallmark’ of autism (Du Bois et al., 2014; Guastella et al., 2010), these claims are often based on indirect evidence—such as impaired theory of mind or a claimed lack of prosocial behaviour—thought to rely on emotion recognition and affect-sharing (Ben Shalom, Belmonte, Gaigg, Bowler, 2021; Stolier et al., 2020). Despite the widespread acceptance of this view, direct studies of socioemotional processing in autism have produced highly mixed findings—for a review see Cuve et al. (2018) or Uljarevic and Hamilton (2013)—suggesting that socioemotional impairments are far from universal in autism.

Appeals to the heterogeneity of autism do not *explain* these mixed findings, rather they just provide a redescription of the variability across autistic individuals (note: we use the word autistic to refer to individuals with autism as this terminology is preferred by the autistic community, Kenny et al., 2016). In contrast, a body of work suggests that heterogeneity with respect to socioemotional processing within the autistic population may be systematic, and explained by co-occurring alexithymia. Alexithymia describes an inability to identify and express one’s emotions (Nemiah, 1976), and is associated with deficits in the recognition of affective information from others (Brewer et al., 2016; Grynberg et al., 2012). Whilst the prevalence of alexithymia is higher in the autistic population (approximately 50%) than in the general population (Bird & Cook, 2013; Cook et al., 2013; Kinnaird et al., 2019; Trevisan et al., 2016), alexithymia and autism have been argued to be distinct. Proponents of this view point out that although approximately 50% of individuals with autism meet criteria to be considered alexithymic, a further 50% do not. Furthermore, the increased prevalence of alexithymia in the autistic population is not specific to autism, but is observed in numerous other psychiatric conditions (Hobson et al., 2019, 2020; Taylor et al., 1996; Westwood et al., 2017). Alexithymia is therefore argued to be neither necessary nor sufficient for an autism diagnosis. In support of the ‘alexithymia hypothesis’—the idea that, where observed, socioemotional deficits in autism are due to co-occurring alexithymia and not autism—several group differences between autistic and neurotypical individuals on socioemotional tasks are no longer evident when alexithymia is controlled for (Bird & Cook, 2013; Bird et al., 2010; Cook et al., 2013; Cuve et al., 2021; Santiesteban et al., 2020; Shah et al., 2016). Conversely, a number of studies have reported dissociable effects of autistic and alexithymic traits on socioemotional abilities in the autistic and general population (Bird et al., 2011; Foulkes et al., 2015; Desai et al., 2019; Mul et al., 2018). Thus, variance with respect to alexithymia in samples of autistic individuals (and those with elevated autistic traits) may explain why socioemotional deficits are, or are not, observed across studies.

For the alexithymia hypothesis to be logically coherent, autism and alexithymia must be distinct. However, others have considered alexithymia to be a symptom or consequence of autism (Gaigg, 2012; Quattrocki & Friston, 2014). Under this view, alexithymia would be yet another characteristic of autism which shows variability within the autistic (and general) population, albeit a characteristic which covaries with socioemotional functioning (such that autistic symptoms causes some individuals to be alexithymic and have poor emotion recognition and low levels of empathy, while other autistic individuals are unaffected in these domains). Understanding whether alexithymia and autism are distinct, or whether alexithymia is a symptom or product of autism is therefore important for theoretical reasons.

There are also clinical reasons to ascertain whether alexithymia and autism are distinct, particularly in relation to autism assessment, diagnosis and treatment. If the emotional difficulties in autism are in fact due to alexithymia, and alexithymia is distinct from autism, then an assessment of alexithymia is required when diagnosing autism to ensure that a full picture of the patient’s strengths and weaknesses is obtained, and their needs addressed. This scenario may also require a rethinking of diagnostic protocols; evidence suggests that alexithymia increases the likelihood of an autism diagnosis at least two-fold (Berthoz & Hill, 2005; Hobson et al., 2020). If alexithymia and autism are indeed separable, then diagnostic protocols may need revision to account for the fact that not all autistic individuals will show socio-emotional problems and yet they may still struggle with restricted interests and communication more broadly. Furthermore, autistic individuals who exhibit good socioemotional functioning (due to an absence of alexithymia) may not be referred for assessment, or receive a diagnosis, if the presence of good socioemotional functioning is deemed to preclude an autism diagnosis.

Autism and alexithymia are operationalised using questionnaires or interviews to identify diagnostic behaviours, symptoms or traits. Two extensively used measures of autism and alexithymia are the AQ-50 (Baron-Cohen et al., 2001) and TAS-20 (Bagby 1994a, 1994b), respectively. The AQ-50 measures autistic traits across a number of dimensions (*social skills, communication, imagination, attention to detail* and *attention switching*), while the TAS-20 measures three facets of alexithymia; *difficulties identifying and describing one’s own emotions*, and *externally oriented thinking*. Both the AQ-50 and TAS-20 were designed to be used with both clinical and non-clinical samples. In order to explore whether alexithymia is a product of autism (i.e. that a common factor underlies autistic and alexithymic traits, where that common factor may be autism itself), or whether autism and alexithymia traits are distinct, we examined the overlap between alexithymic and autistic traits as measured by the TAS-20 and AQ-50. We focused on the measurement level for three reasons: (1) these measures operationalise the constructs into measurable traits; (2) given their frequency of use, potential overlap between these measures has practical implications for research and clinical practice, and (3) they are compatible with prevailing models of autism and alexithymia as traits that exist to varying degrees in the general population. We used two main approaches to examine the overlap between these measures: dimensionality reduction and a network approach.

Dimensionality reduction was addressed with a joint exploratory factor analysis of the AQ-50 and TAS-20, with additional testing of confirmatory, theoretically-driven models of the covariance between dimensions of autism and alexithymia in an independent sample. This approach allows competing models of the relationship between autistic and alexithymic traits (i.e. the common vs distinct latent factor models) to be formally contrasted, however it is not without its problems. Specific issues include problems associated with non-unique or nearly-equivalent model solutions, and the fact that the true underlying model may be different from the factor model (van Bork et al., 2017).

To overcome these problems, we also used a network approach which allows investigation of complex relationships between variables without the assumptions associated with dimension reduction techniques (Epskamp et al., 2017). This approach builds on systems theories of psychopathology, which attempt to explain relationships between different symptoms and the frequent comorbidity seen in psychopathology (Borsboom & Cramer, 2013). Underlying this approach is the view that psychopathology is a dynamic system, where all nodes (symptoms or traits) can influence other nodes in the system (network), and these dependencies can be quantified. For instance, even if completely separable at the latent dimensional level, autism and alexithymia may influence one another causally. To illustrate, difficulties identifying feelings might lead to difficulties socialising, or difficulties with communication might make it difficult to describe feelings, leading to strong dependencies between autistic and alexithymic traits.

In Study 1 we conducted a joint exploratory factor analysis of the AQ-50 and TAS-20 items in a group of neurotypical individuals as well as in a group of clinical participants with autism and other conditions. We also estimated networks using both AQ-50 and TAS-20 items for both groups (N = 931). In Study 2, we used data from 849 new participants to conduct a confirmatory factor and network analysis based on the results of Study 1, before pooling the data for comparable samples across studies (N = 1571) to confirm results. While previous research generally shows a positive association between alexithymic and autistic traits (Kinnaird et al., 2019), the shared variance is often small (less than 30%). Therefore, we hypothesised that both approaches would separate autism and alexithymia, suggesting they are distinct conditions.

## Study 1. Exploratory Factor and Network Analysis

### Methods

#### Participants

Data was gathered from 1138 participants recruited for a larger project. There was an especially large number of non-binary (individuals identifying as neither male or female) autistic participants, a proportion thought to be non-representative of the autistic population as a whole (Murphy et al., 2020). As a consequence, analyses were conducted both with and without this subset of participants, and results were consistent. After accounting for missing data, the full set of participants reported here comprised 931 (50% female) participants, of whom 522 reported no mental health conditions. Of the remaining 409 participants self-reporting a clinical diagnosis, 122 reported a diagnosis of autism, 287 reported another clinical diagnosis (63 depression and anxiety, 34 depression, 22 anxiety, 20 gender dysphoria), and the remaining 148 reported other conditions and combinations of two or more conditions, (e.g., a mix of eating disorders, personality disorders, ADHD, OCD, and substance use). The inclusion of clinical participants, particularly those with autism, ensured that the full range of autism and alexithymia traits was captured. However, while a proportion (approximately 35%) of participants reporting an ASD diagnosis were recruited from a volunteer database with independent confirmation of their diagnosis, the majority of participants were recruited online and their diagnosis could not be confirmed. As the clinical sample was heterogeneous, with autistic people on average reporting three other co-occurring conditions, all clinical participants were grouped together. The average age of the participants was 29 years (SD = 12.03). The clinical group was slightly older (M_age_ = 30.73, SD = 11.29) than the neurotypical group (M_age_ = 28.45, SD = 12.26, t_(717)_ = 2.30, p < 0.02, *d* = 0.19).

### Instruments

#### Autism Spectrum Quotient—AQ-50

The AQ-50 assesses levels of autistic traits. It was originally thought to have five dimensions: *social skills (SS), communication (COM), imagination (IMG), attention to detail (ATD)* and *attention switching (AS).* Items are scored on a four-point scale (maximum score 200 as there are 50 items). Confirmatory studies of the factor structure have been inconclusive, psychometric properties are, however, acceptable (Ruzich et al., 2015). In the current sample, using the original five factor structure, internal reliability ranged from 0.66 to 0.83 for individual subscales, and was 0.89 for the entire scale.

Prior to jointly estimating the factor and network structures for both questionnaires, a confirmatory factor analysis (CFA) was conducted on the AQ-50 to test the original factor structure (Baron-Cohen et al., 2001) as well as several other proposed factor structures (English et al., 2020; Hoekstra et al., 2008). The original five factor structure underperformed compared to more parsimonious solutions (see CFA of Individual Measures in Supplemental Materials), which is consistent with previous reports that the AQ-50 contains redundancies that do not improve measurement precision (Lundqvist & Lindner, 2017). In the total sample the average AQ score was 112.52 (SD = 20.84), with the clinical group reporting higher autistic traits (M = 122, SD = 24.30) than the neurotypical group (M = 108.87, SD = 18.06, t_(720)_ = 13.3, p < 0.001, *d* = 0.61).

#### Toronto Alexithymia Scale—TAS-20

The TAS-20 assesses levels of alexithymic traits. The original structure included three factors: *difficulties identifying feelings* (DIF), *difficulties describing feelings* (DDF) and *externally-oriented thinking* (EOT). Each item is scored on a five-point scale (maximum score 100 as there are 20 items). The psychometric properties of the TAS-20 have been consistently reported as adequate to excellent (Sekely et al., 2018). In the current sample, the internal reliability of the TAS-20 was 0.87 for the total scale and ranged from 0.66 to 0.86 for individual subscales. The CFA on the factor structure of the TAS-20 was best fitted by the originally proposed three-factor solution plus a method factor for reversed items (Bagby et al., 2020; Preece et al., 2020 see CFA of Individual Measures in Supplementary Materials). In the total sample, the average alexithymia score was 49.11 (SD = 12.23), with the clinical group reporting higher levels of alexithymia (M = 53.02, SD = 24.30) than the neurotypical group (M = 47.62, SD = 18.06, t_(720)_ = 5.4, p < 0.001, *d* = 0.25).

### Statistical Analyses

#### Exploratory Factor Analysis (EFA)

An EFA was estimated jointly for both the AQ-50 and TAS-20 using a minimum residual estimation. Because the AQ-50 and TAS-20 items are on different scales, the EFA used the correlation (rather than covariance) matrix.

#### Factor Extraction and Rotation

We used parallel analysis and an oblique—*promax* rotation, motivated by previous positive correlations between TAS-20 and AQ-50 scores (Poquérusse et al., 2018), which was also observed in the current sample (r_(720)_ = 0.62, p < 0.001). Given the large number of variables, 0.4 was used as the threshold for factor loadings. Fit indices (LTI, RMSEA) were used to assess the overall factor solution. Group-specific analyses provided similar results and are included in the Supplementary Materials (EFA Study 1).

#### Assumption Checks

Multivariate normality was assessed by plotting the distribution of all variables. Factorability assumptions were assessed using the Kaiser–Meyer–Olkin (KMO) test and Bartlet test for sphericity (BTS). All items had Measure of Sampling Adequacy (MSA) > 0.5, ranging from 0.6 to 0.98, overall MSA = 0.93. Similarly, the BTS was also significant, Χ^2^_(2415)_ = 24,415.70, p < 0.001. This indicates that the item covariance matrix can be simplified using a reduced number of factors.

#### Network Analyses

In psychological networks, relationships between symptoms or traits are estimated as undirected networks by means of partial correlations between all variables. The following concepts are required for interpretation: nodes, edges and centrality. Symptoms/traits are termed *nodes*, and the connections between these symptoms/traits are termed *edges*. Nodes (symptoms/traits) can be described in terms of their *centrality*, a measure of how strongly connected a node is to all other nodes. Nodes with more connections are more central, and are traditionally understood as critical points of influence on other nodes (i.e. changes in a more central node will affect a greater number of other nodes in comparison to a less connected node). The average centrality indicates the interconnectedness of the network. Edges, the connection between two nodes, can be described in terms of their *strength*, which is the size of the partial correlation between two nodes conditioned on all other nodes. Thus, two nodes that make an edge are dependent after controlling for all other nodes in the network (Epskamp & Fried, 2018).

The main advantage of the network approach over the factor approach is that it offers an alternative to the nearly-equivalent and non-unique factor solution problem (van Bork et al., 2017). Importantly, the Gaussian Graphical Models (GGM) used for estimating undirected networks are typically equivalent to the latent factor approach (Golino & Epskamp, 2017), but are uniquely identified, that is, the underlying ‘true’ parameters of the network can be recovered (Epskamp, 2020). The network approach can therefore provide converging evidence for whether autism and alexithymia are distinct.

#### Network Estimation

We estimated a joint network for AQ-50 and TAS-20 items using a GGM which uses a graphical Lasso regularization method based on Extended Bayesian Information Criteria to minimise spurious connections (Friedman et al., 2008; Epskamp & Fried., 2018). We estimated both clinical and neurotypical networks as well as a joint network with all data. Our goal was not to interpret specific nodes or edges because questionnaires usually include multiple items that tap onto the same dimension. Additionally, the feasibility and validity of specific interpretations with networks of this size are highly debated (Castro et al., 2019; Fried & Cramer, 2017). Instead, we focus on assessing the overall structure of the network to test the central question of whether autism and alexithymia are distinct. To visualise the networks we used the walktrap algorithm which allows detection of clusters of items in exploratory graphical analysis akin to the dimensions of factor analysis (Golino & Epskamp, 2017).

#### Network and Node Description and Inference

The validity of network metrics is dependent upon how stable the network is, since, like any other statistical test, differences may be due to chance and sensitive to statistical power. We bootstrapped 95% confidence intervals (CIs) around edge weights and computed a centrality stability coefficient (CSC). CSC estimates range from 0 to 1, with a CSC > 0.5 indicative of a stable network (Epskamp et al., 2018a, 2018b; Fried et al., 2018). We also conducted edge weights difference tests to compare specific connections, and centrality difference tests to compare centrality metrics within the networks.

In addition, we assessed network centrality based on strength as it is considered to be the most reliable estimate of centrality (Epskamp et al., 2018a, 2018b). In line with recommendations (Haslbeck & Fried, 2017) shared variance of each node with its neighbours was computed using the *mgm* package in R, to assess the absolute level of interconnectedness. This metric can be understood in terms of predictability of the node by other nodes in the network.

#### Network Comparison

To compare networks across different samples, we first computed a similarity measure by correlating the ordered edge weights from both networks (Fried et al., 2018). Second, we used a Network Comparison Test (Van Borkulo & Boschloo, 2017), a permutation-based test which allows comparison of networks on three aspects: network invariance, edge invariance and global strength. The network structure invariance analyses test for a difference in overall structure (rather than individual connections) between two networks. The edge invariance test tests the null hypothesis that all edges are exactly identical in two networks. Edge invariance was tested using Bonferroni-corrected pairwise comparison tests to examine how many edges differed across the networks. The third test—global strength comparison—tests the null hypothesis that both networks have the same degree of absolute interconnectedness. Because of the large number of nodes and edges estimated in the joint network for autism and alexithymia items, which may reduce statistical power, we repeated the three steps above for a network analysis based on factor scores derived using the original factor structures for these questionnaires (see Factor Score Networks in Supplementary Materials) which yielded results consistent with those reported here.

## Results

### Exploratory Factor Analysis

#### Removed Items

For the TAS-20, three items did not reach the factor loading threshold. All items belonged to the EOT subscale. For the AQ-50, 23 items failed to reach the factor loading threshold. The majority belonged to the attention-related factors of the AQ-50 scale (*AS* and *ATD*; see Table S.2 and S.3 in Supplementary Materials for details).

#### Factor Loadings

Factor reduction suggested solutions ranging from 5 to 8 factors, where autism and alexithymia items loaded on entirely separate factors with a final solution of six factors (see Table 1). The first factor contained items assessing *social interests and abilities (SOC)* from the AQ-50 and explained about 9% of the variance. The second factor contained only TAS-20 items focused on *identifying* and *describing feelings and sensations (FEE)* and explained 8% of the variance. The third factor contained items assessing *flexibility (FLX)* in behaviour and interests, mostly consisting of *communication* and *attention switching* items from the AQ-50 and explained 5% of the variance. The fourth factor contained *externally-oriented thinking (EOT)* items from the TAS-20 and explained 4.5% of the variance. The 5th factor exclusively contained items belonging to the *imagination (IMG)* subscale of the AQ-50, and explained 3.6% of the variance. The final factor explained only 2.7% of the variance and contained items belonging to the *attention to detail (ATD)* subscale of the AQ-50.

**Table 1:** Factor loadings

Items	Description	Factor						
		1	2	3	4	5	6	UN
TAS_1	I am often confused about what emotion I am feeling		0.755					0.383
TAS_2	It is difficult for me to find the right words for my feelings		0.769					0.332
TAS_3	I have physical sensations that even doctors don’t understand		0.429					0.756
TAS_4	I am able to describe my feelings easily		0.671					0.402
TAS_6	When I am upset, I don’t know if I am sad, frightened, or angry		0.65					0.509
TAS_7	I am often puzzled by sensations in my body		0.51					0.635
TAS_8	I prefer to just let things happen rather than to understand why they turned out that way				0.451			0.747
TAS_9	I have feelings that I can’t quite identify		0.799					0.404
TAS_10	Being in touch with emotions is essential				0.529			0.642
TAS_11	I find it hard to describe how I feel about people		0.529					0.547
TAS_12	People tell me to describe my feelings more		0.453					0.641
TAS_13	I don’t know what’s going on inside me		0.737					0.393
TAS_14	I often don’t know why I am angry		0.492					0.632
TAS_15	I prefer talking to people about their daily activities rather than their feelings				0.533			0.599
TAS_17	It’s difficult for me to reveal my innermost feelings, even to close friends		0.414					0.596
TAS_18	I can feel close to someone, even in moments of silence				0.404			0.762
TAS_19	I find examination of my feelings useful in solving personal problems				0.492			0.701
AQ_1	I prefer to do things with others rather than on my own	0.506						0.725
AQ_3	If I try to imagine something, I find it very easy to create a picture in my mind					0.602		0.709
AQ_6	I usually notice car number plates or similar strings of information						0.547	0.502
AQ_7	Other people frequently tell me that what I’ve said is impolite, even though I think it is polite			0.501				0.662
AQ_8	When I’m reading a story, I can easily imagine what the characters might look like					0.653		0.586
AQ_11	I find social situations easy	0.836						0.275
AQ_13	I would rather go to a library than a party	0.661						0.636
AQ_14	I find making up stories easy					0.651		0.606
AQ_15	I find myself drawn more strongly to people than to things	0.506						0.629
AQ_16	I tend to have very strong interests which I get upset about if I can’t pursue			0.533				0.644
AQ_17	I enjoy social chit-chat	0.82						0.406
AQ_18	When I talk, it isn’t always easy for others to get a word in edgeways			0.48				0.814
AQ_22	I find it hard to make new friends	0.74						0.497
AQ_23	I notice patterns in things all the time						0.33	0.485
AQ_26	I frequently find that I don’t know how to keep a conversation going	0.546						0.465
AQ_29	I am not very good at remembering phone numbers						0.558	0.71
AQ_34	I enjoy doing things spontaneously	0.547						0.623
AQ_35	I am often the last to understand the point of a joke			0.42				0.713
AQ_38	I am good at social chit-chat	0.787						0.359
AQ_39	People often tell me that I keep going on and on about the same thing			0.611				0.634
AQ_40	When I was young, I used to enjoy playing games involving pretending with other children					0.45		0.761
AQ_44	I enjoy social occasions	0.895						0.362
AQ_46	New situations make me anxious	0.547						0.54
AQ_47	I enjoy meeting new people	0.763						0.471
AQ_49	I am not very good at remembering people’s date of birth						0.522	0.75
AQ_50	I find it very easy to play games with children that involve pretending					0.429		0.774

1: Social skills (SOC); 2: Feelings and sensations (FEE), 3: Flexibility (FLX); 4: Externally oriented thinking (EOT), 5: Imagination (IMG) and 6: Attention to detail (ATD); Uniqueness (UN)

The final solution explained 34% of the variance, and showed an acceptable fit (RMSEA = 0.042, TLI = 0.834). As the first extracted factor explained less than 30% of the total variance, this suggests that the solution does not represent a unidimensional latent measure (Slocum, 2011).

#### Factor Characteristics

**Fig. 1:**
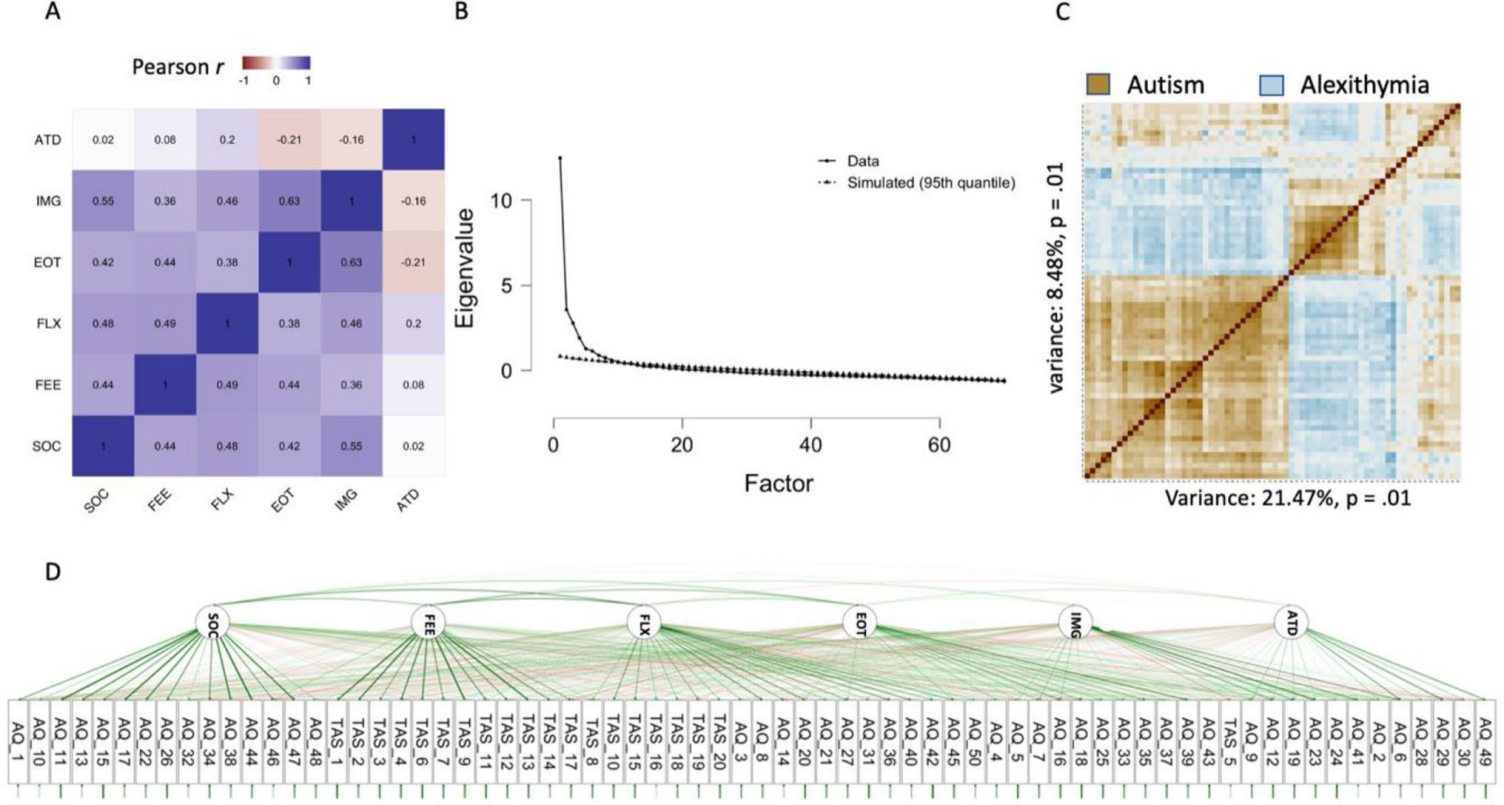
Extracted factors, clusters and factor correlations. **A** Heatmap of factor intercorrelations: most factors showed small to moderate positive correlations, apart from *ATD*. *SOC* social skills; *FEE* feelings and sensations; *FLX* flexibility; *EOT* externally oriented thinking, *IMG* imagination, *ATD* attention to detail. **B** Scree plot of the factor solution. Solid line represents real data, dashed line depicts simulation from parallel analysis suggesting a 5–7 factor solution. **C** A PCA based clustering representation autism and alexithymia traits. **D** Path diagram: strongest connections for each factor contain either autism or alexithymia traits, not both

All extracted factors showed small to strong positive intercorrelations, except for *ATD* which showed small positive to negative correlations with the other factors (see Fig. 1). The presence of correlations that are close to zero, or negative, again suggests that the extracted solution is unlikely to be unidimensional.

#### Reliability Analyses

Internal reliability for the factors was computed separately for both groups (neurotypical and clinical). In the neurotypical sample, for the alexithymia items, reliability scores were: *FEE* α = 0.89 [0.88, 0.91], *EOT* α = 0.64 [0.59, 0.69], and global reliability α = 0.87 [0.88, 0.90]. For the autism subscales, reliability was as follows: *SOC* α = 0.9 [0.89, 0.91], *FLX* α = 0.65 [0.61, 0.7], *IMG* α = 0.67 [0.63, 0.72], *ATD* α = 0.65 [0.6, 0.69], with global reliability α = 0.84 [0.82, 0.86]. The reliability for all items combined (across both scales) was α = 0.9 [0.89, 0.91]. For the clinical group, reliability scores were similar, with alexithymia subscale reliability ranging from 0.50 to 0.91, autism scales ranging from 0.65 to 0.93, and global reliability for both scales α = 0.93 [0.92, 0.95].

### Exploratory Factor Analysis: Results Summary

The results of the exploratory factor analysis were incompatible with the idea that alexithymia is a product of autism or that it reflects the same condition. Results did not support a single latent factor, and alexithymia and autism traits (i.e. TAS-20 and AQ-50 items) loaded onto entirely separate factors. The factor solution was reliable, and had an acceptable fit.

#### Network Estimation

**Fig. 2:**
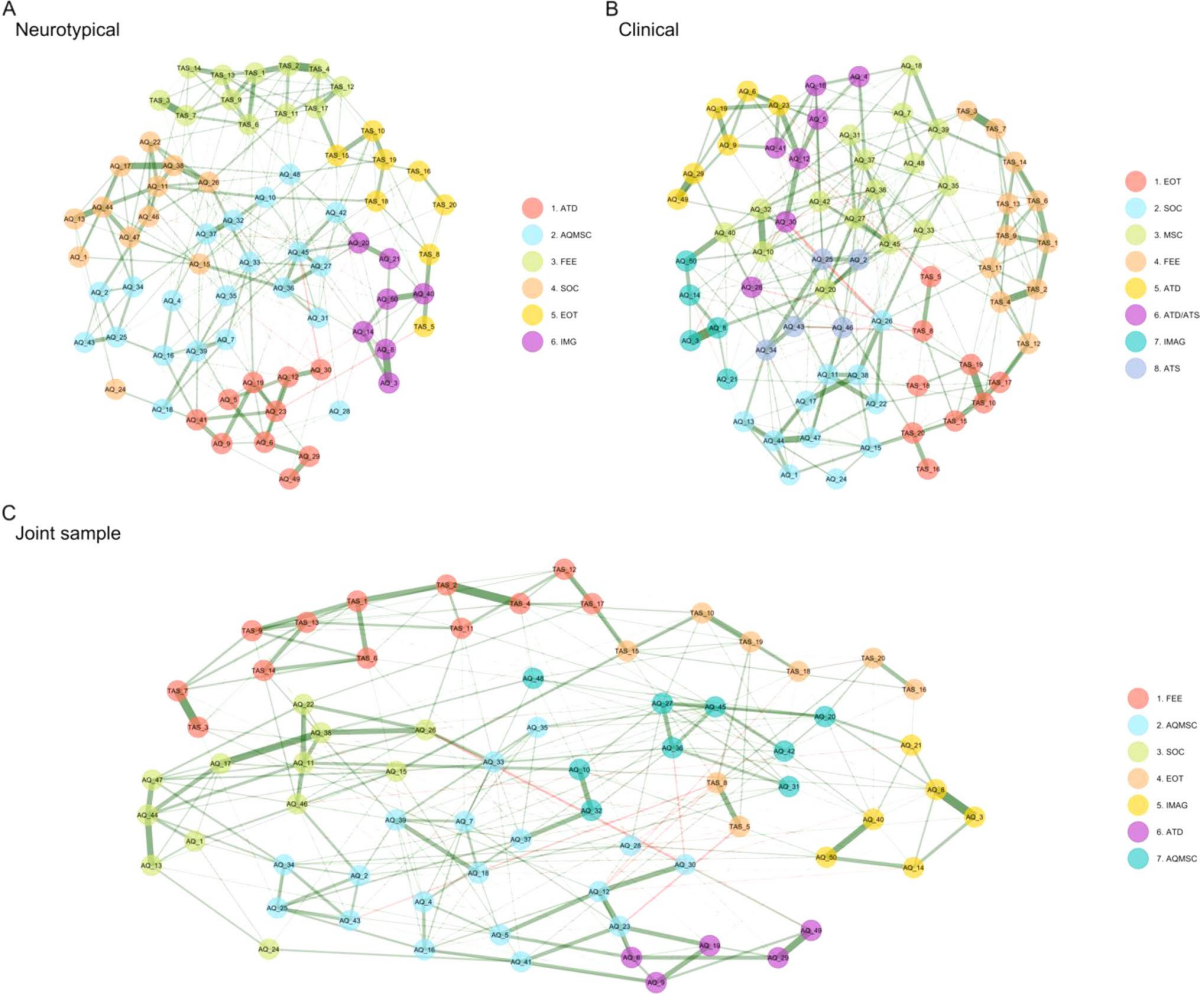
Exploratory graph networks for alexithymia and autism traits. Each colour represents a ‘cluster’ of connected items within the network. All networks separated autism and alexithymia into different clusters, consistent with the results of the Exploratory Factor Analysis. *FEE* feelings and sensations; *AQMSC* miscellaneous autistic traits including social, communication and imagination; *ATD* attention to detail, *ATS* attention switching, *SOC* social skills and interests, *COM* communication; *EOT* externally oriented thinking, *IMAG* imagination

The estimated networks for the neurotypical, clinical and combined groups are visualised in Fig. 2. Descriptively, the estimated networks produced on average seven clusters, which separated autism and alexithymia items in a similar manner to the factor analysis. All networks were largely comparable, and so for brevity we focus on the neurotypical network as it is better powered and less heterogenous, and will be used for replicability analyses in Study 2. In this network, Cluster 1 included mostly *attention to detail* items from the AQ-50. Cluster 2 included AQ-50 items which tended to be those excluded from the final solution in the factor analysis, made up of a mixture of items from *attention switching* to *communication*. Cluster 3 perfectly aligned with the *feelings and sensations* factor extracted in the factor analysis consisting of TAS-20 items only. Similarly, Cluster 4 aligned perfectly with the *social interests and abilities* factor extracted in the factor analysis, made up exclusively of AQ-50 items. Cluster 5 included the *EOT* factor of the TAS-20 and Cluster 6 included the *imagination* traits from the AQ-50. The clusters had no overlap of autism and alexithymia traits, consistent with the suggestion that the two conditions are distinct.

#### Network Stability

We assessed network stability by randomly dropping cases (participants) and nodes (traits) and computing correlation coefficients for centrality indices with the original sample. Our results showed that the neurotypical and jointly-estimated network were reliably estimated, with a CSC of 0.52 and 0.59 respectively, greater than the recommended cut-off of 0.5. However, the clinical network was unstable, with a CSC of 0.13, likely due to reduced statistical power, and therefore we computed a factor network which was sufficiently powered and produced results consistent with those reported above (see Factor Score Network Analysis in Supplementary Materials).

#### Network Comparisons

**Fig. 3:**
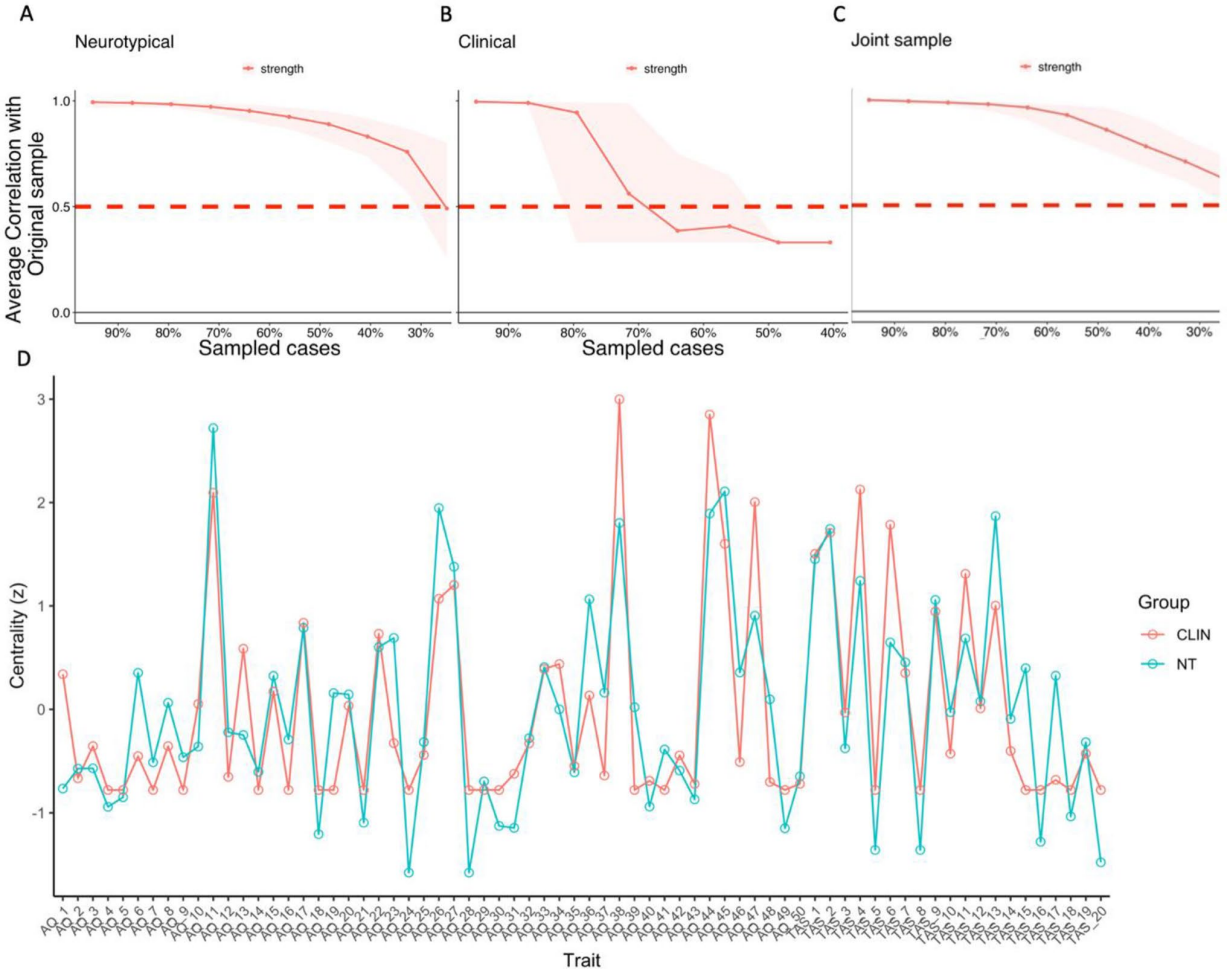
Network stability plots. **A to C** shows the correlation stability coefficient (the average correlation between the full sample and a sub-sample created through resampling—y axis) as a function of the percentage of cases (participants) retained in the sub-sample (x axis). The neurotypical network was more reliable than the clinical network. **D.** Centrality plot showing standardised node strength (the degree of interconnectedness of a trait/symptom). *Clin* clinical sample; *NT* neurotypical sample

Because of the large number of items it is not feasible to focus on interpretation of specific nodes and edges. Instead, centrality estimates were computed and are visualised in Fig. 3. Centrality order was highly correlated across networks, 0.82 for *clinical* vs. neurotypical, and 0.70 for *clinical* vs *joint sample*; and 0.89 for *neurotypical* vs *joint sample*.

This means that the order of the most central (interconnected) items was relatively consistent across networks. Node predictability (an index of network connectivity) was higher in the *clinical* (0.47) than *neurotypical* (0.20) network, with nodes sharing on average 34% of variance (i.e., the amount variance in ratings for each autistic or alexithymic trait, was explained by neighbouring nodes). The correlation between edge weight matrices (the strength of trait connections) was 0.42 for *clinical* vs *joint network*, 0.82 for *neurotypical* vs *joint network* and 0.6 for *clinical* vs. *neurotypical*. These values indicate relatively strong similarity across networks. For the Network Comparison Test, the null hypothesis of structural invariance, that is, that both networks (clinical and neurotypical) are identical, was not rejected (M = 0.25, p = 0.64). There were also no significant differences in global strength between neurotypical and clinical networks (S = 21.84, p = 0.33), indicating that they have a similar degree of interconnectedness. When testing for edge invariance (i.e. that each pair of node connections are equivalent across networks), none of the edges reached significance after Bonferroni correction. As stated above, network estimation for the clinical group and the group comparison may be underpowered given the large number of traits in the network, which also impacts the sensitivity of the NCT. Therefore, we repeated analyses 1 to 3 using factor scores rather than individual items. Overall, the results were consistent (see Factor Score Network—Supplementary Materials). These results suggest that neurotypical and clinical networks can be considered structurally identical, and the separate clustering of alexithymia and autism variables is consistent with the suggestion that they are distinct conditions.

### Network Analysis Results Summary

The results of the network analysis were consistent with the EFA in that autistic and alexithymic traits were separated into distinct clusters, and the nature of those clusters broadly mapped onto the factors identified in the factor analysis. The neurotypical only, clinical, and joint networks were largely comparable, as were networks constructed on factor scores to guard against low statistical power.

## Discussion

Both the factor and network analyses suggested that autistic and alexithymic traits cluster separately, despite being positively correlated. The factor analysis suggested separate factors made up of exclusively autistic or alexithymic traits. The explained variance from each factor, and factor intercorrelations, suggests a multidimensional solution rather than a unitary structure. Networks of both items and factor scores were consistent with the factor analysis and supported strong independence of autism and alexithymia. The results of Study 1 therefore support the claim that autism and alexithymia are distinct.

## Study 2

Study 2 aimed to confirm the factor and network structures estimated in Study 1, specifically the separation of autism and alexithymia dimensions at both a latent level and in terms of the relationship between traits in a joint network. Based on Study 1, for the confirmatory factor analysis we predicted that a factor structure of autistic and alexithymic traits as separate latent causal constructs would fit the data better than a unitary factor structure. For the network analysis, we hypothesised that alexithymic and autistic traits would cluster separately, and expected to replicate the network structure from Study 1.

## Methods

### Participants

A total of 849 (70% female) neurotypical participants completed the AQ-50 and TAS-20 questionnaires (see Methods in Study 1). Participants were on average 28 years old (SD = 9.67) and did not differ significantly from the neurotypical sample in Study 1 in terms of age (t_(1362)_ = 0.455, p = 0.65), or alexithymia scores (t_(1369)_ = 1.23, p = 0.22). Study 2 participants (M = 111, SD = 17.47) scored slightly higher than neurotypical participants in Study 1 (t_(1369)_ = − 2.84, p = 0.005, d = -0.16) on the AQ-50, but lower than the clinical group in Study 1 (t_(1069)_ =  − 0.6.33, p < 0.001, d = 0.52). TAS-20 and AQ-50 showed a medium-sized positive correlation (r_(847)_ = 0.48, p < 0.001), lower than in Study 1 (z = − 3.62, p < 0.001).

#### Confirmatory Factor Analyses

A confirmatory factor analysis was conducted in R using the lavaan package (v.0.6–6).

**Fig. 4:**
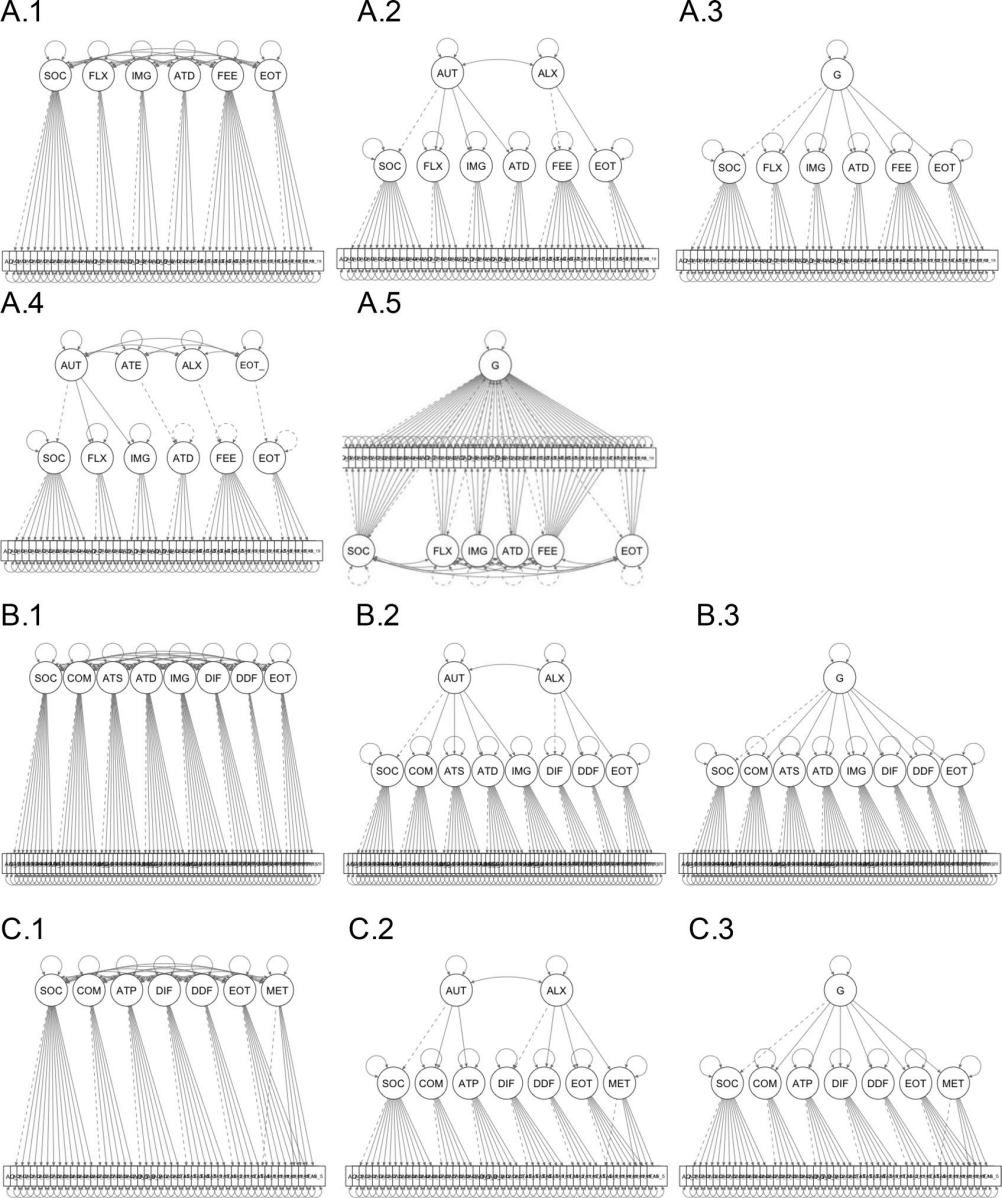
Graphical representation of confirmatory models. Representation of the models fitted in the confirmatory factor analysis of the AQ-50 and TAS-20. **A** models were based on Exploratory Factor Analysis solution in Study 1, **B** models were based on the original factor structures of each questionnaire and **C** models were based on proposed alternative solutions to the original factor structures

Eight models were fit to distinguish between unitary or distinct factor structure(s) underlying autistic and alexithymic traits as measured by the AQ-50 and the TAS-20, respectively. Full model details are given in Supplementary Materials (Confirmatory Factor Analysis: Model Specification) but three families of models were tested (see Fig. 4).

Models in Group A were based on the six-factor solution obtained in the joint factor analysis of the AQ-50 and TAS-20 in Study 1. Models in Group B were based on the original factor structures for each questionnaire (five factor solution for the AQ-50: *social skills*, *communication*, *imagination*, *attention to details* and *attention switching* (Baron-Cohen et al., 2001), and 3-factor solution for TAS-20: *difficulties identifying feelings*, *difficulties describing feelings* and *externally oriented thinking* (Bagby et al., 1994a, 1994b). Models in Group C were based on the best performing factor solutions identified in meta-analyses and reviews (English et al., 2020), which were also the best preforming models for the individual scales in Study 1 (see CFA of Individual Measures—Supplementary Materials). The fitted models included three factors for the AQ (*social*, *communication* and *attention*), and four factors for the TAS-20 (*DIF, DDF, EOT* and a method factor for reversed items; Preece et al., 2020; Watters et al., 2016). Within each family of models, the following models were compared: (1) distinct correlated factors; (2) autism factors and alexithymia factors are driven by distinct latent causes (i.e. autism and alexithymia, respectively); or (3) a common latent factor gives rise to autism and alexithymia.

#### Model Assessment and Comparison

Fit indices including CFI, TLI and RMSEA were used to assess model properties. A Likelihood Ratio Test was used to compare nested models and AIC and BIC were used in addition to fit indices for non-nested models.

#### Network Analysis

Network analysis was conducted as in Study 1. To confirm the results of Study 1, the network obtained in Study 1 was compared to that obtained using data from Study 2, and also confirmed using data pooled across Studies (N = 1571).

## Results

### Confirmatory Factor Analysis

Results were consistent across all three model families. In each, the best performing model was the one in which the factors of autism and alexithymia were separate, or with separate latent causal factors. Summary statistics of model comparisons (where appropriate) are summarised here, and full details of fit indices and model comparisons are provided in Supplementary Materials (Table S.4).

From Group A the best performing model was Model A.1, which contained a six-factor correlated solution. The model fit was acceptable (CFI = 0.80, and RMSEA of 0.05 90% CI (0.53, 0.57), p < 0.001). Neither model A.2, (*χ*^2^_(8)_ = 99.53, *p* < 0.001) nor A.3 provided a better fit to the data (*χ*^2^_(9)_ = 104.44, *p* < 0.001). Model A.4 showed poor fit and model A.5 failed to converge. From Group B, the best performing model (B.1) specified distinct correlated factors for autism and alexithymia, as opposed to second-order models (B.2 and B.3). However, Group B models showed poor fit indices and were therefore not considered further. From Group C, model C.1, specifying a seven-factor solution with no higher-order terms, was the best performing model. This model showed acceptable fit with CFI of 0.841, RMSEA of 0.50 [0.48, 0.52], ns. Model C.1 outperformed model C.3 (*χ*^2^_(14)_ = 141.83, p < 0.001). Model C.2 showed negative variances and therefore was not considered further.

For Model A.1 (and all best-fitting models in each group), all items showed significant positive factor loadings, with standardized coefficients ranging from 0.1 to 0.83 (see Table S.3 in Supplementary Materials). There were also significant positive correlations among 4 of the 6 factors (*social skills and interests*, *feelings and sensations*, *flexibility* and *imagination*, ranging from 0.11 to 0.48). This indicates that participants’ scores are likely to correlate positively on those dimensions. However, as shared variance for all cases is less than 20%, it suggests that there is little overlap in the measured constructs. In sum, these results support the proposal that autism and alexithymia are distinct.

### Confirmatory Network Analysis

#### Network Estimation

**Fig. 5:**
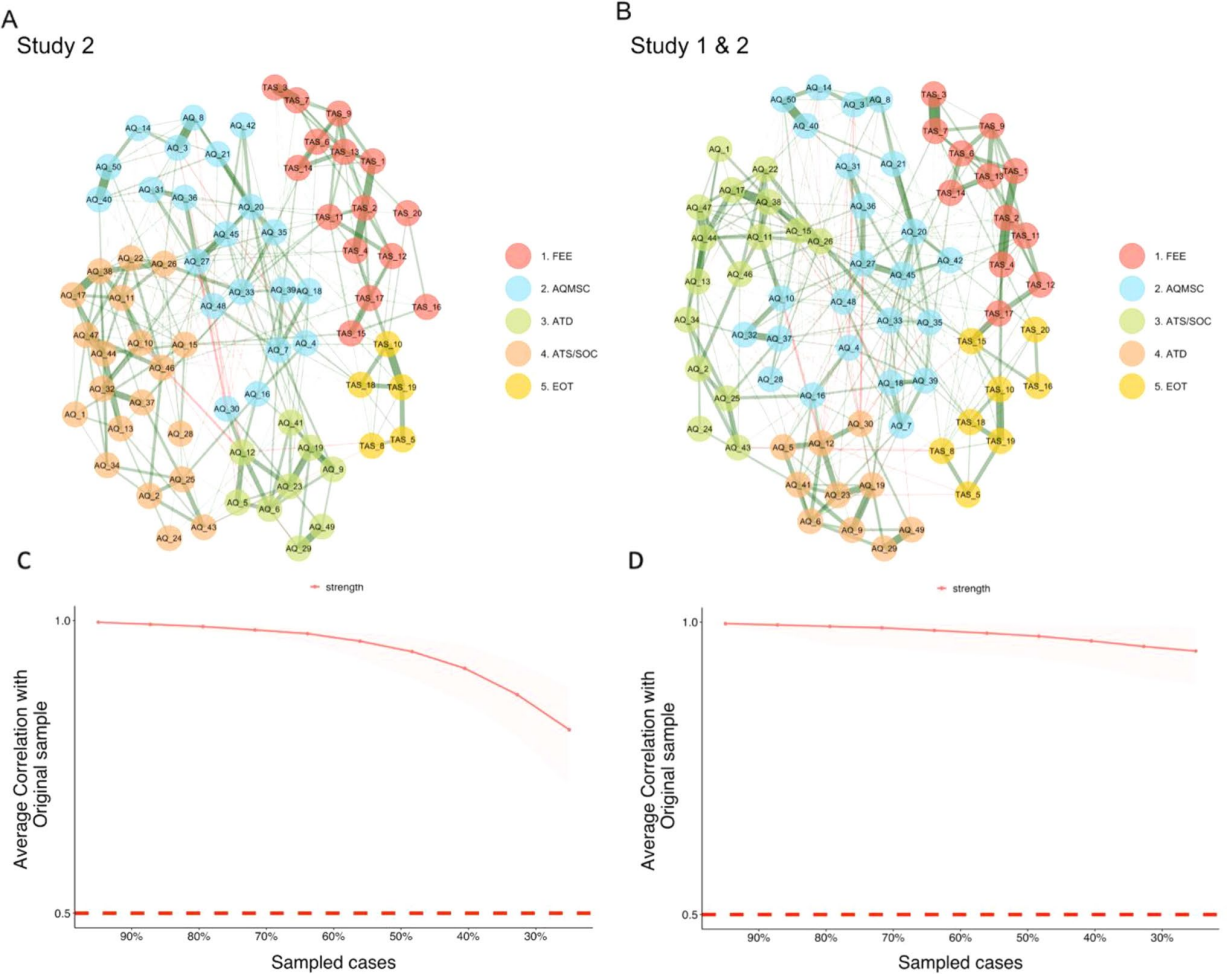
Estimated networks—Study 2. Network from study 2 (**A**) and pooled network combining neurotypical samples from studies 1 and 2 (**B**). Each colour represents a cluster of traits. **C** and **D** show the correlation stability coefficient—the correlation of centrality indices between the full sample and sub-samples across various sub-sample sizes. Values > 0.5 suggest stable and reliable networks. *FEE* feelings and sensations; *AQMSC* miscellaneous autistic traits including social, communication and imagination; *ATD* attention to detail; *ATS* attention switching; *SOC* social skills and interests, *EOT* externally oriented thinking

Figure 5 depicts the network estimated in Study 2, and the jointly-estimated network derived from Studies 1 and 2. As can be seen, in Study 2, as in Study 1, autism and alexithymia items clustered separately. There were five clusters identified in Study 2, rather than the 6 identified in Study 1. Nonetheless, alexithymia clusters were mostly the same as in Study 1, with *feelings and sensations (FEL)* items clustering together (Cluster 1). However, unlike in Study 1, three EOT items clustered with the *FEL* items, with the rest of the EOT items clustering together (Cluster 5). AQ items produced three clusters: Cluster 2 contained a mixture of social, communication scales and imagination items, Cluster 3 consisted mainly of the *attention-related* items identified in Study 1, and Cluster 4 mostly contained items assessing *social skills*.

Overall, alexithymia and autism clusters showed positive correlations, within and between clusters, but the autism cluster contained some negative correlations from the autism miscellaneous Cluster 2 to the attention-related Cluster 3. The neurotypical network from Studies 1 and 2 also produced a similar network. Across studies, alexithymia clusters were more consistent than autism clusters.

#### Network Inference and Stability

Centrality measures were in general consistent with Study 1 and are presented in Supplementary Materials (Network Analysis—Study 2). Network bootstrapping demonstrated that the network was stable with a CSC of 0.67 (greater than the recommended value of 0.5).

#### Network Comparison

There was a strong similarity between the network structures from Study 1 and 2, with a correlation between edge weight matrices of 0.83. The NCT indicated no significant differences in network invariance (structure) M = 0.16, p = 0.37, nor in global strength (the average strength of the connections) S = 3.78, p = 0.84. There was also only 1 significant difference in edge invariance in the network (less than 1%). Together, these results suggest that the estimated networks are largely similar. As in Study 1, networks estimated using factors were also consistent with a separation of alexithymia and autism (see Study 2—Factor Score Networks in Supplementary Materials).

## Discussion

Study 2 confirmed that alexithymic and autistic traits are best characterised as distinct. In the CFA, models specifying a common latent structure fit the data poorly. Of note is that models that defined separate higher-order factors (of autism and alexithymia) did not fit the data significantly better than a model with no higher order factors (i.e. where each sub-factor of the AQ-50 and TAS-20 was independent). Results of the network analysis replicated across studies; autism and alexithymia traits again clustered separately, supporting the claim that autism and alexithymia are distinct conditions.

## General Discussion

Socioemotional difficulties have long been considered a hallmark of autism (APA, 2013; Guastella et al. 2010; Du Bois et al. 2014), but it has recently been argued that any socioemotional difficulties in the autistic population are caused by co-occurring alexithymia (Bird & Cook, 2013). For this account to be logically coherent, autism and alexithymia must be distinct conditions, yet it has been claimed that alexithymia is a product of autism (Quattrocki & Friston, 2014, Gaigg, 2012; Ben Shalom et al., 2021). In this series of studies we therefore sought to examine whether alexithymia should be considered a consequence of autism, or distinct from it. Results support the argument that alexithymia and autism are distinct. Study 1 used factor analytic and network approaches to assess responses to the most widely-used self-report measures of autism and alexithymia and found distinct autism and alexithymia factors and clusters. Study 2 used confirmatory methods to show that all models assigning a unitary latent factor common to autistic and alexithymic traits fitted the data poorly in comparison to both multidimensional models and a model specifying distinct latent sources of covariance for autism and alexithymia factors. Network analyses again supported the independence of autism and alexithymia.

The results from studies 1 and 2 are consistent with previous reports showing double dissociations between effects of autism and alexithymia (Bird et al., 2011; Bernhardt et al., 2014; Desai et al., 2019; Mul et al., 2018). The independence of autism and alexithymia has important implications for research and clinical practice. For research, results suggest the need to rethink models that attempt to account for emotional difficulties in autism without considering the role of alexithymia. Although autism and alexithymia are not the same construct, the increased prevalence of alexithymia in autism may be crucial for understanding increased vulnerability to emotional problems (e.g., poor emotion regulation) in autism. For clinical practice, our results suggest a need for assessment of socio-emotional abilities in general, and alexithymia specifically, when working with autistic individuals.

The use of both clinical and non-clinical participants ensures that the full range of scores for alexithymia and autism are captured, which reduces the consequences of bias associated with selecting samples based on diagnostic scores which tends to result in a restricted range of scores in variables of interest and is problematic for factor and network approaches (Maric et al., 2004; De Ron et al., 2021). The assessment of the AQ suggests that the measurement of autistic traits needs improvement. While the key dimensions of alexithymia were reliably identified across analyses, the same was not true for autism using the AQ, a finding consistent with previous studies (English et al., 2020).

Our focus on the measurement level in this study (using the AQ-50 and TAS-20) represented a practical solution to the conceptual problem of potential autism/alexithymia overlap, but it could be considered a limitation of the study. Rather than using self-report questionnaires, symptom/trait severity could be assessed using diagnostic interviews or performance on objective tests. Ideally, future studies will be better powered to explore whether the network structures in clinical and autistic groups are similar to neurotypical samples. Additionally, future research could benefit from novel developments using generalized network psychometric models that account for latent influences on networks, given that most of psychopathology measurement is based on measurement of latent factors (Epskamp et al., 2017).

Another potential limitation is our use of a cross-sectional adult sample in this study. Future studies could use dynamic network models based on longitudinal data which could inform causal models of how autistic and alexithymic symptoms are related (Epskamp et al., 2018a, 2018b). Developmental studies of this kind would be especially useful, allowing the relationship between alexithymic and autistic traits to be tracked over time. Such work would also allow the exploration of the multiple possible developmental routes for alexithymia outlined by Hobson et al (2019), particularly whether alexithymia may be causally related to language impairments in a sub-sample of individuals.

It should also be acknowledged that the clinical sample included in this study was not exclusively of autistic individuals with an independently-verified diagnosis, and included a higher proportion of female participants than might be expected in such a sample. The average IQ of the samples included in the study may also be considered to be not representative of the autistic population as a whole (Chiang et al., 2014; Bishop et al., 2015). As such, it is possible that results may differ in a representative sample of individuals diagnosed with autism. In the absence of such evidence, however, the current results provide evidence for the independence of autism and alexithymia traits.

## Conclusion

Across two studies and using factor analytic and network analyses we show that alexithymia and autism are distinct, though they frequently co-occur. Consideration of alexithymia is therefore likely to aid research into the socioemotional abilities of individuals with autism, and to contribute to diagnosis and treatment in clinical practice.

## Supplementary Information

Below is the link to the electronic supplementary material.

Supplementary file1 (DOCX 23176 kb)

## Data Availability

https://osf.io/5fn8b/.
