# Supplementary material for: Are Autistic and Alexithymic Traits Distinct? A Factor-Analytic and Network Approach
Source: J Autism Dev Disord. 2021 Jun 1;52(5):2019–34. doi: 10.1007/s10803-021-05094-6 (PMC9021140; doi:10.1007/s10803-021-05094-6)
Supplement: Supplementary file 1 — Supplementary file1 (DOCX 23176 kb) [file 10803_2021_5094_MOESM1_ESM.docx]

**Supplementary Materials**

**Are Autistic and Alexithymic Traits Distinct? A Factor-Analytic and Network Approach**

*Table S.1*

Descriptive Measures for Study 1 & 2

|  | **Study1** | | **Study2** | |  |  |
| --- | --- | --- | --- | --- | --- | --- |
|  | **N = 522** | | **N = 849** | |  |  |
| Variable | **Mean** | **SD** | **Mean** | **SD** | **t** | **p** |
| Age | 28.46 | 12.28 | 28.19 | 9.67 | 0.46 | ns |
| DIF | 16.20 | 5.72 | 15.01 | 6.09 | 3.60 | *** |
| EOT | 18.20 | 4.28 | 18.74 | 4.72 | -2.14 | * |
| DDF | 13.22 | 4.53 | 13.05 | 4.77 | 0.68 | ns |
| SS | 20.75 | 5.66 | 21.57 | 5.45 | -2.69 | ** |
| AS | 24.17 | 4.75 | 24.74 | 4.48 | -2.24 | * |
| ATD | 24.59 | 4.98 | 24.87 | 5.23 | -0.98 | ns |
| COM | 20.18 | 5.06 | 20.34 | 5.18 | -0.56 | ns |
| IMG | 19.20 | 4.45 | 20.16 | 4.70 | -3.74 | *** |
| TAS-20 | 47.62 | 11.57 | 46.80 | 12.49 | 1.22 | ns |
| AQ-50 | 108.88 | 18.06 | 111.68 | 17.47 | -2.84 | ** |

Notes. *DIF =* *Difficulties identifying feelings, EOT = Externally oriented thinking; DDF = Difficulties describing feelings; SS = Social skills; ATD = Attention to detail; AS = Attention switching; COM = Communication; IMG* = *Imagination; TAS-20 = Toronto Alexithymia Scale -20; AQ-50 = Autism Spectrum Quotient Scale - 50* * < .05, ** < .01, *** < .001. Note this data if from the neurotypical participants used for replication comparisons in study 2.

**Study 1**

**Exploratory Factor Analysis (EFA) – Study 1**

*Table S.2*

List of all AQ-50 and TAS-20 items

| Item | Description |
| --- | --- |
| TAS_1 | I am often confused about what emotion I am feeling. |
| TAS_2 | It is difficult for me to find the right words for my feelings. |
| TAS_3 | I have physical sensations that even doctors don’t understand. |
| TAS_4 | I am able to describe my feelings easily. |
| TAS_5* | I prefer to analyse problems rather than just describe them. |
| TAS_6 | When I am upset, I don’t know if I am sad, frightened, or angry. |
| TAS_7 | I am often puzzled by sensations in my body. |
| TAS_8 | I prefer to just let things happen rather than to understand why they turned out that way. |
| TAS_9 | I have feelings that I can’t quite identify. |
| TAS_10 | Being in touch with emotions is essential. |
| TAS_11 | I find it hard to describe how I feel about people. |
| TAS_12 | People tell me to describe my feelings more. |
| TAS_13 | I don’t know what’s going on inside me. |
| TAS_14 | I often don’t know why I am angry. |
| TAS_15 | I prefer talking to people about their daily activities rather than their feelings. |
| TAS_16* | I prefer to watch “light” entertainment shows rather than psychological dramas. |
| TAS_17 | It’s difficult for me to reveal my innermost feelings, even to close friends. |
| TAS_18 | I can feel close to someone, even in moments of silence |
| TAS_19 | I find examination of my feelings useful in solving personal problems. |
| TAS_20* | Looking for hidden meanings in movies or plays distracts from their enjoyment. |
| AQ_1 | I prefer to do things with others rather than on my own. |
| AQ_2* | I prefer to do things the same way over and over again. |
| AQ_3 | If I try to imagine something, I find it very easy to create a picture in my mind. |
| AQ_4* | I frequently get so strongly absorbed in one thing that I lose sight of other things. |
| AQ_5* | I often notice small sounds when others do not. |
| AQ_6 | I usually notice car number plates or similar strings of information. |
| AQ_7 | Other people frequently tell me that what I’ve said is impolite, even though I think it is polite. |
| AQ_8 | When I’m reading a story, I can easily imagine what the characters might look like. |
| AQ_9* | I am fascinated by dates. |
| AQ_10* | In a social group, I can easily keep track of several different people’s conversations. |
| AQ_11 | I find social situations easy. |
| AQ_12* | I tend to notice details that others do not. |
| AQ_13 | I would rather go to a library than a party. |
| AQ_14 | I find making up stories easy. |
| AQ_15 | I find myself drawn more strongly to people than to things. |
| AQ_16 | I tend to have very strong interests which I get upset about if I can’t pursue. |
| AQ_17 | I enjoy social chit-chat. |
| AQ_18 | When I talk, it isn’t always easy for others to get a word in edgeways. |
| AQ_19* | I am fascinated by numbers. |
| AQ_20 | When I’m reading a story, I find it difficult to work out the characters’ intentions. |
| AQ_21 | I don’t particularly enjoy reading fiction. |
| AQ_22 | I find it hard to make new friends. |
| AQ_23 | I notice patterns in things all the time. |
| AQ_24 | I would rather go to the theatre than a museum. |
| AQ_25 | It does not upset me if my daily routine is disturbed. |
| AQ_26 | I frequently find that I don’t know how to keep a conversation going. |
| AQ_27* | I find it easy to “read between the lines” when someone is talking to me. |
| AQ_28* | I usually concentrate more on the whole picture, rather than the small details. |
| AQ_29 | I am not very good at remembering phone numbers. |
| AQ_30* | I don’t usually notice small changes in a situation, or a person’s appearance. |
| AQ_31* | I know how to tell if someone listening to me is getting bored. |
| AQ_32* | I find it easy to do more than one thing at once. |
| AQ_33* | When I talk on the phone, I’m not sure when it’s my turn to speak. |
| AQ_34 | I enjoy doing things spontaneously. |
| AQ_35 | I am often the last to understand the point of a joke. |
| AQ_36* | I find it easy to work out what someone is thinking or feeling just by looking at their face. |
| AQ_37* | If there is an interruption, I can switch back to what I was doing very quickly. |
| AQ_38 | I am good at social chit-chat. |
| AQ_39 | People often tell me that I keep going on and on about the same thing. |
| AQ_40 | When I was young, I used to enjoy playing games involving pretending with other children. |
| AQ_41* | I like to collect information about categories of things (e.g. types of car, types of bird, types of train, types of plant, etc.). |
| AQ_42* | I find it difficult to imagine what it would be like to be someone else. |
| AQ_43* | I like to plan any activities I participate in carefully. |
| AQ_44* | I enjoy social occasions. |
| AQ_45 | I find it difficult to work out people’s intentions. |
| AQ_46 | New situations make me anxious. |
| AQ_47 | I enjoy meeting new people. |
| AQ_48 | I am a good diplomat. |
| AQ_49 | I am not very good at remembering people’s date of birth. |
| AQ_50 | I find it very easy to play games with children that involve pretending. |

Note. * denotes excluded items in the joint EFA.

*Table S.3*

Factor Loadings for Joint EFA Study 1

|  | | | | | | | | | | | | | |
| --- | --- | --- | --- | --- | --- | --- | --- | --- | --- | --- | --- | --- | --- |
|  | | **Factor 1** | | **Factor 2** | | **Factor 3** | | **Factor 4** | | **Factor 5** | | **Uniqueness** | |
| TAS_1 |  |  |  | 0.894 |  |  |  |  |  |  |  | 0.359 |  |
| TAS_2 |  |  |  | 0.839 |  |  |  |  |  | 0.413 |  | 0.307 |  |
| TAS_3 |  |  |  | 0.540 |  |  |  |  |  |  |  | 0.663 |  |
| TAS_4 |  |  |  | 0.713 |  |  |  |  |  | 0.484 |  | 0.332 |  |
| TAS_5 |  |  |  |  |  |  |  |  |  |  |  | 0.915 |  |
| TAS_6 |  |  |  | 0.778 |  |  |  |  |  |  |  | 0.454 |  |
| TAS_7 |  |  |  | 0.658 |  |  |  |  |  |  |  | 0.583 |  |
| TAS_8 |  |  |  |  |  |  |  |  |  |  |  | 0.848 |  |
| TAS_9 |  |  |  | 0.900 |  |  |  |  |  |  |  | 0.386 |  |
| TAS_10 |  |  |  |  |  |  |  |  |  |  |  | 0.678 |  |
| TAS_11 |  |  |  | 0.625 |  |  |  |  |  |  |  | 0.486 |  |
| TAS_12 |  |  |  | 0.489 |  |  |  |  |  | 0.426 |  | 0.617 |  |
| TAS_13 |  |  |  | 0.849 |  |  |  |  |  |  |  | 0.415 |  |
| TAS_14 |  |  |  | 0.619 |  |  |  |  |  |  |  | 0.610 |  |
| TAS_15 |  |  |  |  |  |  |  |  |  |  |  | 0.675 |  |
| TAS_16 |  |  |  |  |  |  |  |  |  |  |  | 0.887 |  |
| TAS_17 |  |  |  |  |  |  |  |  |  | 0.439 |  | 0.645 |  |
| TAS_18 |  |  |  |  |  |  |  |  |  |  |  | 0.863 |  |
| TAS_19 |  |  |  |  |  |  |  |  |  |  |  | 0.751 |  |
| TAS_20 |  |  |  |  |  |  |  |  |  |  |  | 0.888 |  |
| AQ_1 |  | 0.642 |  |  |  |  |  |  |  |  |  | 0.663 |  |
| AQ_2 |  |  |  |  |  |  |  |  |  |  |  | 0.761 |  |
| AQ_3 |  |  |  |  |  |  |  |  |  |  |  | 0.839 |  |
| AQ_4 |  |  |  |  |  |  |  |  |  |  |  | 0.767 |  |
| AQ_5 |  |  |  |  |  |  |  |  |  |  |  | 0.708 |  |
| AQ_6 |  |  |  |  |  |  |  | 0.640 |  |  |  | 0.578 |  |
| AQ_7 |  |  |  |  |  |  |  |  |  |  |  | 0.711 |  |
| AQ_8 |  |  |  |  |  | 0.490 |  |  |  |  |  | 0.754 |  |
| AQ_9 |  |  |  |  |  |  |  | 0.533 |  |  |  | 0.705 |  |
| AQ_10 |  |  |  |  |  |  |  |  |  |  |  | 0.640 |  |
| AQ_11 |  | 0.823 |  |  |  |  |  |  |  |  |  | 0.275 |  |
| AQ_12 |  |  |  |  |  |  |  | 0.598 |  |  |  | 0.609 |  |
| AQ_13 |  | 0.713 |  |  |  |  |  |  |  |  |  | 0.575 |  |
| AQ_14 |  |  |  |  |  |  |  |  |  |  |  | 0.833 |  |
| AQ_15 |  | 0.538 |  |  |  |  |  |  |  |  |  | 0.599 |  |
| AQ_16 |  |  |  |  |  |  |  |  |  |  |  | 0.709 |  |
| AQ_17 |  | 0.832 |  |  |  |  |  |  |  |  |  | 0.417 |  |
| AQ_18 |  |  |  |  |  |  |  |  |  |  |  | 0.820 |  |
| AQ_19 |  |  |  |  |  |  |  | 0.547 |  |  |  | 0.686 |  |
| AQ_20 |  |  |  |  |  | 0.633 |  |  |  |  |  | 0.616 |  |
| AQ_21 |  |  |  |  |  |  |  |  |  |  |  | 0.940 |  |
| AQ_22 |  | 0.686 |  |  |  |  |  |  |  |  |  | 0.496 |  |
| AQ_23 |  |  |  |  |  |  |  | 0.712 |  |  |  | 0.483 |  |
| AQ_24 |  |  |  |  |  |  |  |  |  |  |  | 0.879 |  |
| AQ_25 |  |  |  |  |  |  |  |  |  |  |  | 0.674 |  |
| AQ_26 |  | 0.542 |  |  |  |  |  |  |  |  |  | 0.462 |  |
| AQ_27 |  |  |  |  |  | 0.684 |  |  |  |  |  | 0.479 |  |
| AQ_28 |  |  |  |  |  |  |  |  |  |  |  | 0.843 |  |
| AQ_29 |  |  |  |  |  |  |  |  |  |  |  | 0.903 |  |
| AQ_30 |  |  |  |  |  |  |  |  |  |  |  | 0.851 |  |
| AQ_31 |  |  |  |  |  | 0.435 |  |  |  |  |  | 0.773 |  |
| AQ_32 |  |  |  |  |  | 0.409 |  |  |  |  |  | 0.698 |  |
| AQ_33 |  |  |  |  |  |  |  |  |  |  |  | 0.602 |  |
| AQ_34 |  | 0.593 |  |  |  |  |  |  |  |  |  | 0.596 |  |
| AQ_35 |  |  |  |  |  |  |  |  |  |  |  | 0.710 |  |
| AQ_36 |  |  |  |  |  | 0.622 |  |  |  |  |  | 0.545 |  |
| AQ_37 |  |  |  |  |  |  |  |  |  |  |  | 0.663 |  |
| AQ_38 |  | 0.786 |  |  |  |  |  |  |  |  |  | 0.315 |  |
| AQ_39 |  |  |  |  |  |  |  |  |  |  |  | 0.662 |  |
| AQ_40 |  |  |  |  |  | 0.424 |  |  |  |  |  | 0.783 |  |
| AQ_41 |  |  |  |  |  |  |  | 0.561 |  |  |  | 0.660 |  |
| AQ_42 |  |  |  |  |  | 0.512 |  |  |  |  |  | 0.694 |  |
| AQ_43 |  |  |  |  |  |  |  |  |  |  |  | 0.815 |  |
| AQ_44 |  | 0.900 |  |  |  |  |  |  |  |  |  | 0.316 |  |
| AQ_45 |  |  |  |  |  | 0.636 |  |  |  |  |  | 0.463 |  |
| AQ_46 |  | 0.491 |  |  |  |  |  |  |  |  |  | 0.615 |  |
| AQ_47 |  | 0.810 |  |  |  |  |  |  |  |  |  | 0.406 |  |
| AQ_48 |  |  |  |  |  |  |  |  |  |  |  | 0.677 |  |
| AQ_49 |  |  |  |  |  |  |  |  |  |  |  | 0.962 |  |
| AQ_50 |  |  |  |  |  |  |  |  |  |  |  | 0.804 |  |
|  | | | | | | | | | | | | | |
| *Note.*  Applied rotation method is promax. Factor 1: *Social skills* *(SOC);* Factor 2: *Feelings and sensations* *(FEE),* Factor 3: *Flexibility and Imagination* *(FLX);* Factor 4: *Externally oriented thinking* *(EOT)*, Factor 5: *Attention to detail* *(ATD).* | | | | | | | | | | | | | |


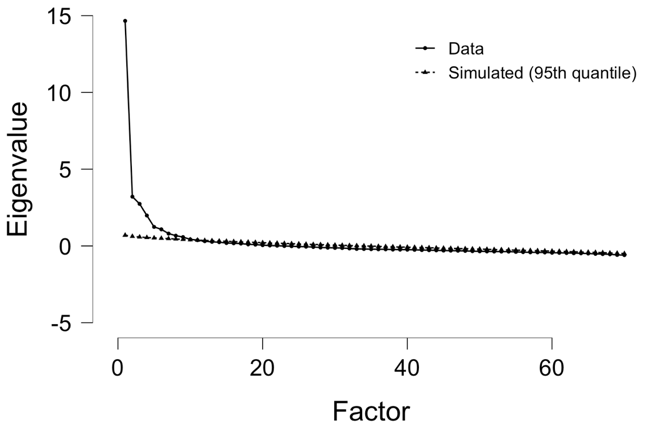


*Figure S.1.* Scree plot of the joint sample EFA

**Network Analysis – Study 1**

**Analysis of all Items**

*Centrality*

A few *social skills* (autism) and *feelings and sensation* (alexithymia) items showed the highest centrality, indicating that these items were strongly connected with other terms in the network (e.g. AQ_11, AQ_45, AQ_26, AQ_44, TAS_13, AQ_38, TAS_2, TAS_1). Conversely, a mix of *attention to detail* (autism), *imagination* (autism), and *externally oriented thinking* (alexithymia) items had standardised centrality below zero, indicating that they were very weekly connected nodes (e.g. AQ_24, AQ_28, TAS_20, TAS_5, TAS_8, TAS16, AQ_18, AQ_49).

1. Neurotypical B. Clinical


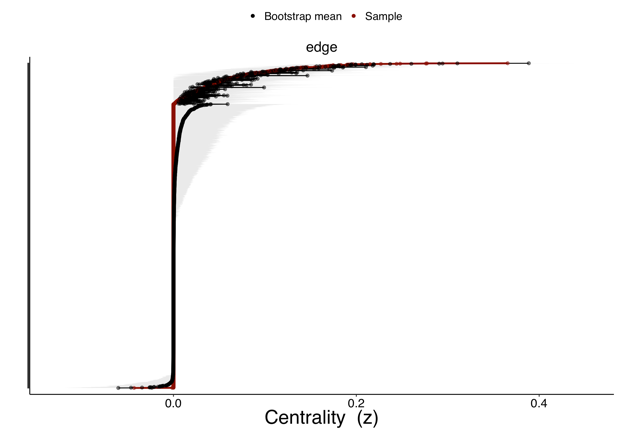

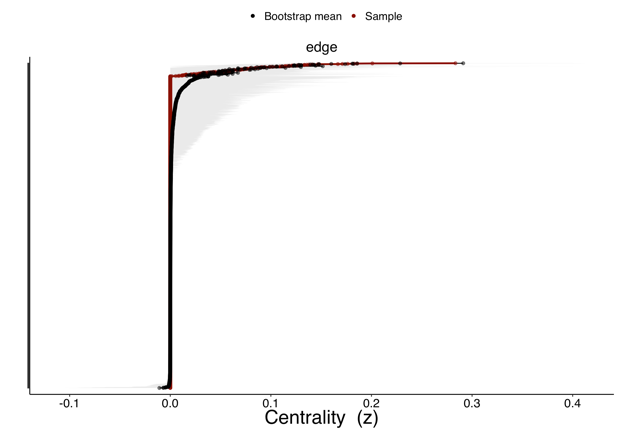


*Figure S.2.* Bootstrapped confidence intervals for edge strength for neurotypical and clinical groups respectively. Edges with CI including zero are not different.

**Factor Score Network Analysis**

*Network Estimation*

The estimated networks using factor scores are visualised in Figure S.3. As in the analyses with all items, the stronger connections appear within autism and alexithymia factors, with generally positive correlations across these clusters. Both networks are highly similar with the strongest edges emerging between difficulties describing and identifying feelings (DDF - DIF) with a regularised partial correlation (RPC) of .47 for neurotypicals and .51 for clinical participants, whereas all other RCPs between DDF or DIF and autism factors were < .15. Similarly, communication and social skills (COM – SOC) showed the strongest RCP: .42 and .44 for the neurotypical and clinical groups respectively. Notably, one edges is slightly stronger in the neurotypical sample network, namely EOT – IMG (RCP = .21) compared to the clinical sample (RCP = .12).

*Network Inference*

Centrality metrics are displayed in Figure S.3.C. The correlation between centrality measures in ordered nodes was generally high, .81 for clinical *vs.* neurotypical networks, .96 for clinical *vs.* full sample, and .92 for NT vs. full sample. Whereas *communication* (COM) and *social skills* (SOC) were the most central factor traits in both networks, *difficulties identifying feelings* was more central in the neurotypical network compared to the clinical network. *Attention to detail* (ATD), *externally oriented thinking* (EOT) and *imagination* (IMG) were consistently low in centrality.

1. Neurotypical B. Clinical C


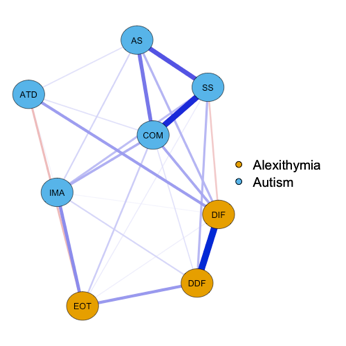

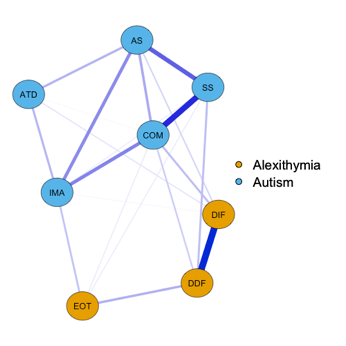

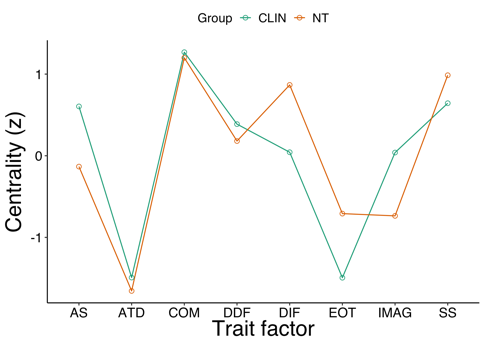


*Figure S.3.* Estimated networks using factor scores. A & B. Estimated network using factor trait scores. C. Standardised centrality for all factors traits. As in the network based on individual items, the node predictability for factors was relatively high, wing neighbouring nodes sharing approximately 41% of the variance for the NT group, and 52% for the clinical group. Node predictability was very strongly correlated with centrality (.94 for NT and .99 for the clinical groups).

*Network Stability*

The CSC was .75 for the NT group and .67 for the clinical group, both values above the recommended .50 cut-off. This indicates that the network structures were robust and stable (see Figure S.4). *Social and* *communication* (SOC-COM) and *difficulties identifying and describing feelings* (DIF – DFF) showed significantly stronger connections than all other edges apart from *social skills* and *communication* (SOC-COM) which were also highly related (p < .05).

A. Neurotypical B. Clinical


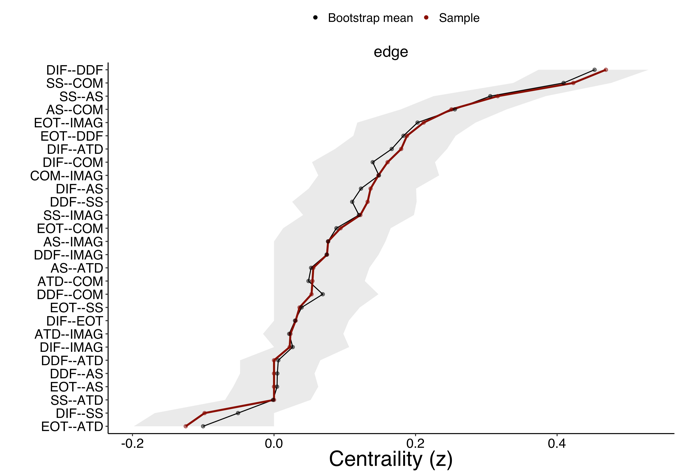

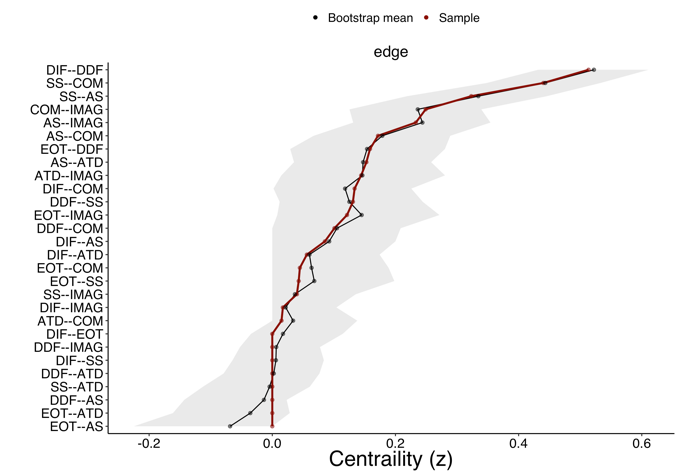


C D


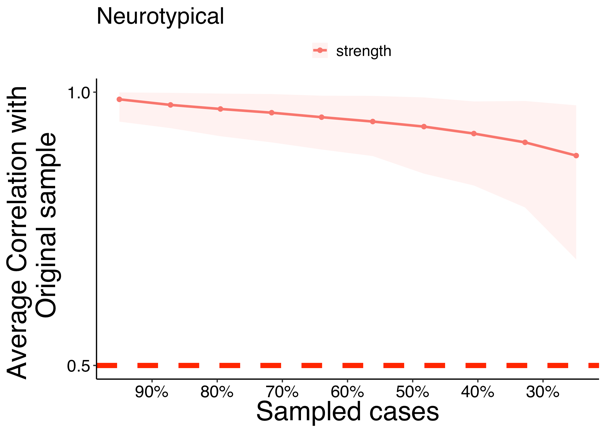

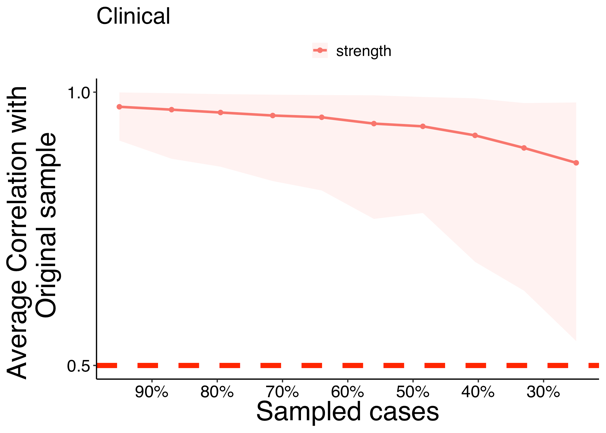


E F


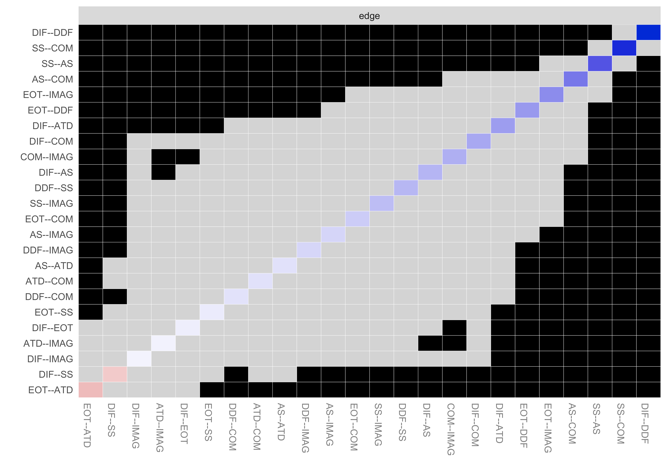

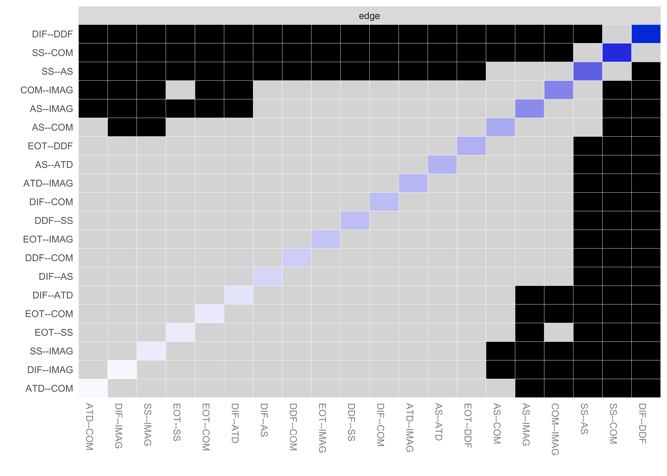


*Figure S.4.* Network stability and inference. A & B: 95% bootstrapped CI around edge weights. Overlapping pairs CIs and CIs including zero are not significant. C & D: the correlation stability coefficient (y axis) per dropped cases (x axis). All network were estimated reliably. E & F: Edge difference plot, comparing edge pairs (strength of pairwise trait connections). Darker squares represent significant (p <.05) difference between the pairwise edge comparisons. Within alexithymia and autism trait connections are significantly stronger than cross alexithymia autism connections.

*Network Comparison*

Edge weights were highly similar across networks with very high correlations (NT – CLIN = .86, NT – full sample = .97 and CLIN vs full sample = .91). The network invariance test was significant (M = 0.295, p < .001) but there were no differences in global strength (Strenght_NT_ = 2.944286, Strenght_CLIIN_ = 3.141381; S = 0.197, p = .63) nor in individual edges at p <.05 (Bonferroni corrected). These results suggest that networks are similar.

**Study 2**

*Confirmatory Factor Analysis: Model Specification*

***Model A:*** Four versions of this model were tested based on the results of Study 1 which indicated a six factor solution: *social skills (SOC),* *feelings and sensations (FEL),* *flexibility (FLX );* *externally oriented thinking* *(EOT),* *imagination (IMG),* *attention to detail (ATD).*

*Model A.1*. Included only the six factors with no second-order factors. This model tests the hypothesis that the facets (factors) of autism and alexithymia are distinct.

*Model A.2*. Included the six factors and two second-order terms for alexithymia and autism. This model tests the hypothesis that while autistic traits and alexithymia may be correlated, they reflect separate underlying latent dimensions.

*Model A.3.* Included the six factors and one higher order factor. This model tests the hypothesis that all autism and alexithymia traits are a product of a single casual latent factor (which may be autism itself).

*Model A.4.* Similar to A.1, but *ATD* and *EOT* were included as separate second order-factors, as these items cluster less consistently than other aspects of autism and alexithymia.

*Model A.5.* Was a bi-factor model, a variation of the general models, where a general factor explains the variance of observed trait symptoms orthogonally to specific factors.

**Model B.** Based on the factor structures proposed by the questionnaire authors - the 5 factor solution for the AQ-50: *social skills*, *communication*, *imagination*, *attention to details* and *attention switching* (Baron-Cohen et al. 2001) and 3-factor solution for TAS-20: *difficulties identifying feelings*, *difficulties describing feelings* and *externally oriented thinking* (Bagby, Parker, et al. 1994). Three versions of this model were fitted.

*Model B.1*. Included the individual factors (8) and no second-order factors.

*Model B.2.* In addition to the first-order terms in B.1, two correlated second-order factors for autism and alexithymia were included.

Model B.3. A model specifying a common latent second-order factor underlying all alexithymia and autism factors.

Given that previous literature suggests that the original factor solutions for the TAS-20 and AQ-50 often underperform in comparison to alternative models (as also observed in Study 1), we fitted additional models (Model C) using the best performing alternative solutions identified in meta-analyses and review studies (English et al. 2020). These were also the best preforming models of the individual scales in Study 1 (see CFA of Individual Measures - below). The fitted models included three factors for the AQ (*social*, *communication* and *attention patterns*), and four factors for the TAS-20 (*DIF, DDF, EOT and a method factor for reversed items*) (Preece et al. 2020; Watters et al. 2016).

*Model C.1.* This model specified a correlation between all 6 factors and no second-order factor (like A1 and B1).

*Model C.2.* Included two second-order correlated factors for autism and alexithymia.

*Model C.3.* Included one single latent common second-order factor*.*

An illustration of the fitted models is visualised in Figure 4 in the manuscript.

**Table S.4.**

*Confirmatory Factor Analysis: Fit indices and model comparisons*

| Model | df | AIC | BIC | X^2^ | X^2^diff | DF | p>X^2^ | RMSEA | CFI | CTI |
| --- | --- | --- | --- | --- | --- | --- | --- | --- | --- | --- |
| MODEL A |  |  |  |  |  |  |  |  |  |  |
| Model A.1 | 887 | 99783.15 | 100271.8 | 3169.427 | NA | NA | ^NA^ | .055[.53,.57]^***^ | .81 | .79 |
| Model A.2 | 893 | 99870.02 | 100330.2 | 3268.297 | 98.87 | 6 | ^***^ | .56[.54,.58]^***^ | .79 | .79 |
| Model A.3 | 895 | 99866.68 | 100317.4 | 3268.957 | 0.66 | 2 | ^ns^ | .56[.54,.58]^***^ | .79 | .79 |
| ModelA.4 | 896 | 99869.59 | 100315.5 | 3273.872 | 4.91 | 1 | ^*^ | .56[.54,.58]^***^ | .79 | .79 |
| Model A.5 | 849 | 99673 | 100342 | 2983.6 | 1 |  |  | .054[.52,.057] ^***^ | .81 | .79 |
|  |  |  |  |  |  |  |  |  |  |  |
| MODEL B |  |  |  |  |  |  |  |  |  |  |
| Model B.1 | 2249 | 155524.2 | 156311.7 | 7973.631 | NA | NA | ^NA^ | .055[.053, .56]^***^ | .67 | .66 |
| Model B.2 | 2268 | 155574.1 | 156271.5 | 8061.51 | 87.88 | 19 | ^**^ | .055[.054, .56]^***^ | .67 | .66 |
| Model B.3 | 2269 | 156041 | 156733.6 | 8530.41 | 468.90 | 1 | ^***^ | .057[.056, .058]^***^ | .64 | .63 |
|  |  |  |  |  |  |  |  |  |  |  |
| MODEL C |  |  |  |  |  |  |  |  |  |  |
| Model C.1 | 964 | 103680.4 | 104235.5 | 2977.54 | NA | NA | ^NA^ | .050[.048,.052]^ns^ | .85 | .83 |
| Model C.2 | 977 | 103718 | 104211.3 | 3041.05 | 63.51 | 13 | ^***^ | .050[.048,.052]^ns^ | .84 | .83 |
| Model C.3 | 978 | 103794.3 | 104282.9 | 3119.364 | 78.31 | 1 | ^***^ | .051[.049,.053]^ns^ | .84 | .83 |

Notes. df – Degrees of freedom for the model; AIC and BIC – Akaike and Bayesian information criteria, respectively. These can be used to compare models directly (lower value models are preferred). X^2^ – Chi-square test for each model solution; X^2^ diff, is the difference in chi-square relative to the nested model, this comparison is only valid for nested models. RMSEA = Root Mean Squared Error of Approximation, the p value indicates whether RMSEA is below the recommended cut-off. CFI (Comparative Fit Index) and TLI (Tucker-Lewis Index) are goodness of fit measures, values >.8 are generally considered acceptable. * < .05, ** < .01, *** < .001

*Table S.5.*

Descriptive statistics for all items

| Variable | Mean | SD | min | max |
| --- | --- | --- | --- | --- |
| AQ1 | 2.46 | 0.90 | 1 | 4 |
| AQ3 | 1.86 | 0.90 | 1 | 4 |
| AQ6 | 2.40 | 1.09 | 1 | 4 |
| AQ7 | 1.75 | 0.94 | 1 | 4 |
| AQ8 | 1.88 | 0.89 | 1 | 4 |
| AQ11 | 2.37 | 0.96 | 1 | 4 |
| AQ12 | 2.95 | 0.94 | 1 | 4 |
| AQ13 | 2.21 | 1.02 | 1 | 4 |
| AQ14 | 2.37 | 1.02 | 1 | 4 |
| AQ15 | 2.20 | 0.89 | 1 | 4 |
| AQ16 | 2.50 | 0.95 | 1 | 4 |
| AQ17 | 2.26 | 0.95 | 1 | 4 |
| AQ18 | 2.03 | 0.93 | 1 | 4 |
| AQ22 | 2.27 | 0.98 | 1 | 4 |
| AQ23 | 2.53 | 0.98 | 1 | 4 |
| AQ26 | 2.32 | 0.98 | 1 | 4 |
| AQ29 | 2.45 | 1.06 | 1 | 4 |
| AQ34 | 2.11 | 0.89 | 1 | 4 |
| AQ35 | 1.88 | 0.93 | 1 | 4 |
| AQ38 | 2.27 | 0.98 | 1 | 4 |
| AQ39 | 2.08 | 0.98 | 1 | 4 |
| AQ40 | 1.89 | 0.94 | 1 | 4 |
| AQ44 | 1.92 | 0.89 | 1 | 4 |
| AQ46 | 2.88 | 0.93 | 1 | 4 |
| AQ47 | 2.11 | 0.92 | 1 | 4 |
| AQ49 | 2.49 | 1.11 | 1 | 4 |
| AQ50 | 2.23 | 1.02 | 1 | 4 |
| TAS1 | 2.40 | 1.18 | 1 | 5 |
| TAS2 | 2.72 | 1.31 | 1 | 5 |
| TAS3 | 1.72 | 1.09 | 1 | 5 |
| TAS4 | 2.72 | 1.18 | 1 | 5 |
| TAS6 | 2.27 | 1.24 | 1 | 5 |
| TAS7 | 2.00 | 1.16 | 1 | 5 |
| TAS8 | 2.27 | 1.12 | 1 | 5 |
| TAS9 | 2.38 | 1.24 | 1 | 5 |
| TAS10 | 1.98 | 1.00 | 1 | 5 |
| TAS11 | 2.35 | 1.20 | 1 | 5 |
| TAS12 | 2.25 | 1.29 | 1 | 5 |
| TAS13 | 2.16 | 1.22 | 1 | 5 |
| TAS14 | 2.09 | 1.24 | 1 | 5 |
| TAS15 | 2.81 | 1.23 | 1 | 5 |
| TAS17 | 3.00 | 1.44 | 1 | 5 |
| TAS18 | 2.12 | 1.09 | 1 | 5 |
| TAS19 | 2.34 | 1.12 | 1 | 5 |
| SOC | 27.37 | 7.42 | 12 | 48 |
| ATD | 12.81 | 3.20 | 5 | 20 |
| FLX | 10.24 | 2.89 | 5 | 20 |
| IMG | 10.23 | 3.05 | 5 | 20 |
| FEL | 28.06 | 9.84 | 12 | 59 |
| EOT | 11.51 | 3.46 | 5 | 23 |

**Joint Confirmatory Factor Analysis of Autism and Alexithymia**

*Table S.5.*

Factor Loadings

| **Factor** | **item** | **est** | **se** | **z** | **p** | **std (all)** |
| --- | --- | --- | --- | --- | --- | --- |
| Social | AQ_1 | 1 | 0 |  |  | 0.327 |
| Social | AQ_11 | 2.49 | 0.269 | 9.239 | < .001 | 0.765 |
| Social | AQ_13 | 1.516 | 0.193 | 7.842 | < .001 | 0.437 |
| Social | AQ_15 | 1.551 | 0.186 | 8.347 | < .001 | 0.516 |
| Social | AQ_17 | 2.452 | 0.266 | 9.224 | < .001 | 0.759 |
| Social | AQ_22 | 2.185 | 0.244 | 8.944 | < .001 | 0.656 |
| Social | AQ_26 | 2.19 | 0.245 | 8.943 | < .001 | 0.656 |
| Social | AQ_34 | 1.27 | 0.164 | 7.739 | < .001 | 0.423 |
| Social | AQ_38 | 2.748 | 0.293 | 9.369 | < .001 | 0.83 |
| Social | AQ_44 | 2.294 | 0.248 | 9.234 | < .001 | 0.763 |
| Social | AQ_46 | 1.606 | 0.193 | 8.314 | < .001 | 0.51 |
| Social | AQ_47 | 2.27 | 0.248 | 9.152 | < .001 | 0.729 |
| Feelings | TAS_1 | 1 | 0 |  |  | 0.759 |
| Feelings | TAS_2 | 1.167 | 0.049 | 23.829 | < .001 | 0.799 |
| Feelings | TAS_3 | 0.534 | 0.043 | 12.371 | < .001 | 0.436 |
| Feelings | TAS_4 | 0.806 | 0.045 | 17.779 | < .001 | 0.614 |
| Feelings | TAS_6 | 0.934 | 0.047 | 19.705 | < .001 | 0.674 |
| Feelings | TAS_7 | 0.664 | 0.045 | 14.665 | < .001 | 0.513 |
| Feelings | TAS_9 | 1.045 | 0.047 | 22.22 | < .001 | 0.751 |
| Feelings | TAS_11 | 0.872 | 0.046 | 18.97 | < .001 | 0.651 |
| Feelings | TAS_12 | 0.783 | 0.05 | 15.618 | < .001 | 0.544 |
| Feelings | TAS_13 | 0.994 | 0.046 | 21.53 | < .001 | 0.73 |
| Feelings | TAS_14 | 0.806 | 0.048 | 16.786 | < .001 | 0.582 |
| Feelings | TAS_17 | 0.757 | 0.057 | 13.328 | < .001 | 0.468 |
| Flexibility | AQ_7 | 1 | 0 |  |  | 0.587 |
| Flexibility | AQ_16 | 0.578 | 0.08 | 7.199 | < .001 | 0.336 |
| Flexibility | AQ_18 | 0.782 | 0.085 | 9.154 | < .001 | 0.467 |
| Flexibility | AQ_35 | 0.643 | 0.081 | 7.982 | < .001 | 0.384 |
| Flexibility | AQ_39 | 1.034 | 0.101 | 10.27 | < .001 | 0.585 |
| EOT | TAS_8 | 1 | 0 |  |  | 0.293 |
| EOT | TAS_10 | 2.062 | 0.299 | 6.894 | < .001 | 0.68 |
| EOT | TAS_15 | 1.33 | 0.23 | 5.778 | < .001 | 0.356 |
| EOT | TAS_18 | 1.734 | 0.263 | 6.583 | < .001 | 0.522 |
| EOT | TAS_19 | 2.205 | 0.321 | 6.861 | < .001 | 0.648 |
| Imagination | AQ_3 | 1 | 0 |  |  | 0.62 |
| Imagination | AQ_8 | 0.938 | 0.082 | 11.406 | < .001 | 0.588 |
| Imagination | AQ_14 | 0.798 | 0.085 | 9.378 | < .001 | 0.436 |
| Imagination | AQ_40 | 0.658 | 0.077 | 8.572 | < .001 | 0.389 |
| Imagination | AQ_50 | 0.941 | 0.089 | 10.557 | < .001 | 0.514 |
| ATD | AQ_6 | 1 | 0 |  |  | 0.686 |
| ATD | AQ_12 | 0.732 | 0.062 | 11.807 | < .001 | 0.583 |
| ATD | AQ_23 | 0.868 | 0.071 | 12.218 | < .001 | 0.661 |
| ATD | AQ_29 | 0.38 | 0.06 | 6.324 | < .001 | 0.268 |
| ATD | AQ_49 | 0.202 | 0.061 | 3.303 | < .001 | 0.136 |

Note. est = estimate; se = standard error; z = standardised estimate; p = p value

*Table S.6*

Latent Factor Correlations for Autism and Alexithymia Joint CFA

| **Factor 1** | **Factor 2** | **Correlation** | **p** |
| --- | --- | --- | --- |
| SOC | FEL | 0.4 | < .001 |
| SOC | FLEX | 0.242 | < .001 |
| SOC | EOT | 0.31 | < .001 |
| SOC | IMG | 0.488 | < .001 |
| SOC | ATD | 0.111 | 0.009 |
| FEL | FLEX | 0.396 | < .001 |
| FEL | EOT | 0.38 | < .001 |
| FEL | IMG | 0.212 | < .001 |
| FEL | ATD | 0.128 | 0.003 |
| FLEX | EOT | 0.263 | < .001 |
| FLEX | IMG | 0.232 | < .001 |
| FLEX | ATD | 0.371 | < .001 |
| EOT | IMG | 0.343 | < .001 |
| EOT | ATD | 0.076 | 0.124 |
| IMG | ATD | -0.094 | 0.063 |

*Note. SOC = Social skills; FEL = Feelings and sensations; FLEX = Flexibility; EOT = Externally oriented thinking; IMG = Imagination; ATD = Attention to detail.*

**Network Analysis – Study 2**

**Analysis on all items**

*Network Inference*

Centrality metrics are plotted in Figure S.5. Centrality order was strongly related for both Study 1 & 2 neurotypical samples, with a correlation of .87. One *feelings and sensation* item and a few *social skills*, *communication* and *imagination* items from the AQ showed the highest centrality strength, indicating that these items were highly interconnected with other items in the network (e.g. TAS_2, AQ_38, AQ_20, AQ_44, AQ_27). On the other hand, a mix of attention to detail and imagination (autism), and EOT (alexithymia) items had standardised centrality below zero, indicating that they were very weekly connected nodes (e.g. AQ_24, TAS_16, AQ_28, TAS_20, TAS_8, TAS_5, AQ_14).

Node predictability for Study 2 sample was .35, that is, there was on average 35% of shared variance between neighbour nodes which highlights the level influence on one node on another nodes assuming that all other nodes are connected to it. This was an increase of 7% compared to the NT network estimated in Study 1. For the full sample combining both Study 1 (NT sample only) and Study 2, the results were similar (38% of shared variance) which suggests that the difference in sample size did not affect the reliability of predictability estimates drastically. Similar to study 1, there was a strong correlation between node centrality and the predictability (. 92) that is the more connected a node is, the more predictable it was. This was similar for the full network estimation with both samples combines (.87).

A B

Study 2 Study 1 & 2


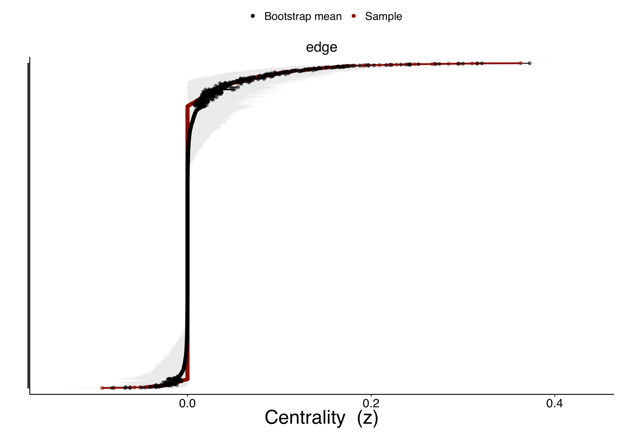

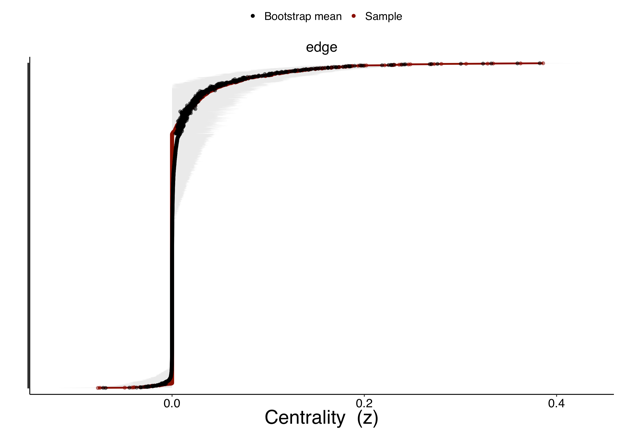


C


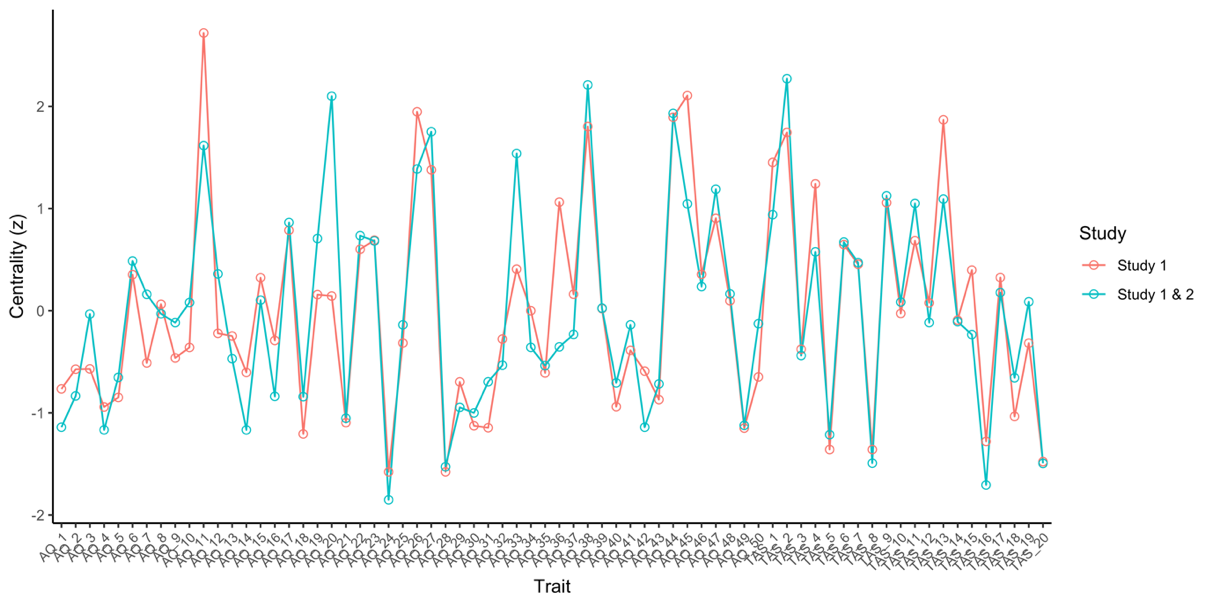


*Figure S.5.* A & B show the bootstrapped CIs around edge weights. Plots C shows z standardised centrality strength for all items.

**Study 2 - Factor Networks**

*Network Estimation*

The estimated networks based on factor scores are displayed in Figure S.6. As in Study 1, we observed strong connections between the alexithymia nodes DIF – DDF (RCP = .51), and DDF-EOT (RPC = .25), compared to connections to autism nodes (RCP < .1). Within the autism nodes, replicating Study 1, we see strong connections between COM-SS (RPC = .47), and COM-AS (RPC = .28), with ATD showing the weakest connections to other nodes (< .1). This pattern was also consistent with the joint network combining Study 1 and Study 2 neurotypical samples; we observed strong edge weights within TAS and AQ networks.

A B C

Study 2 Study 1 & 2


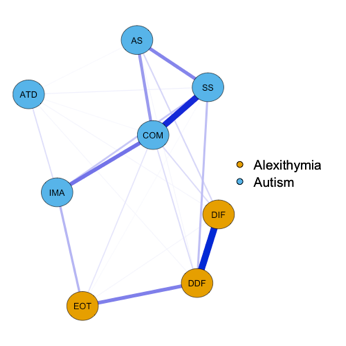

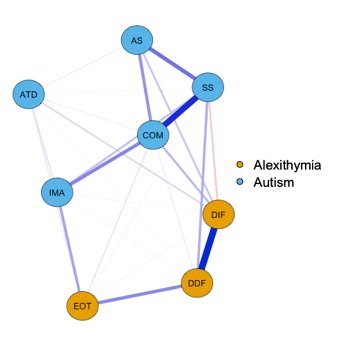

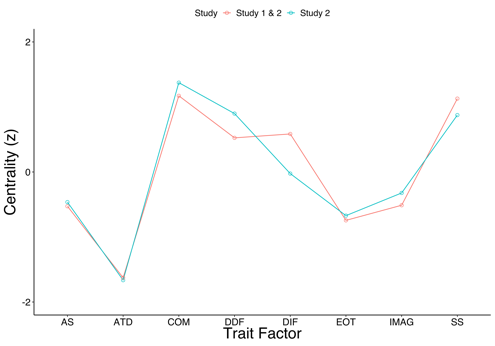


*Figure S.6.* Estimated networks based on factor scores for Study 2 (A) and the neurotypical samples from Studies 1 & 2 (N = 1371) - B. C. Similar to Study 1, communication, social skills (autism) and difficulties with feelings (alexithymia) show the highest centrality (highly connected nodes).

*Network Inference*

Node centrality is visualised in Figure S.6.C. Node centrality was highly related in both Study 1 & 2 networks with a correlation of .88. As in Study 1, communication appeared as the most central factor in the network followed by difficulties describing feelings and social skills. Conversely, attention to detail was the least central node followed by externally oriented thinking. The averaged shared variance between neighbouring nodes was 38% for the Study 2 sample and 13% for the full sample. As in Study 1, there was a strong correlation between node predictability and how central (connected) a node was. The correlation was .98 for the study 2 sample and .68 for the joint network.

*Network stability*

The Study 2 network was stable, with a CSC of .75, and the joint network had a CSC of .74. Trait connections within alexithymia and autism are significantly stronger than between alexithymia autism connections (see Figure S.7.A & B). As in Study 1, DIF – DDF and SS – COM edges had the highest strength, differing significantly from all other edges but not differing from each other (see Figure S.7- E & F).

1. Study 2 B. Study 1 & 2


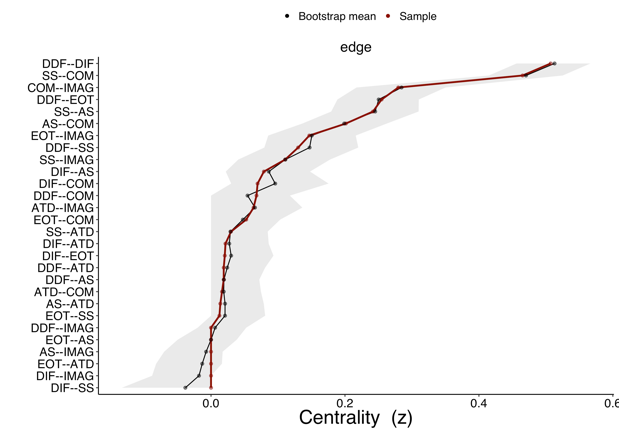

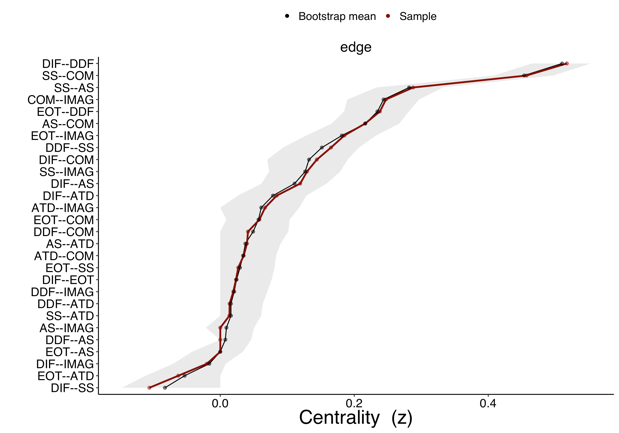


C D


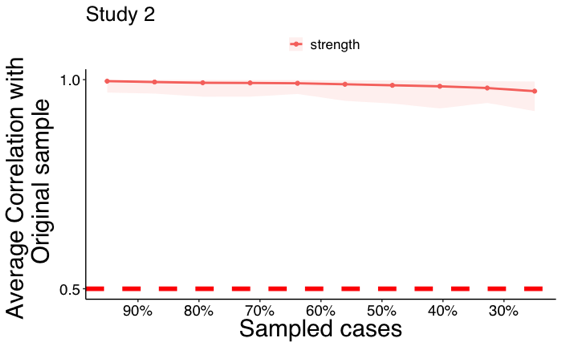

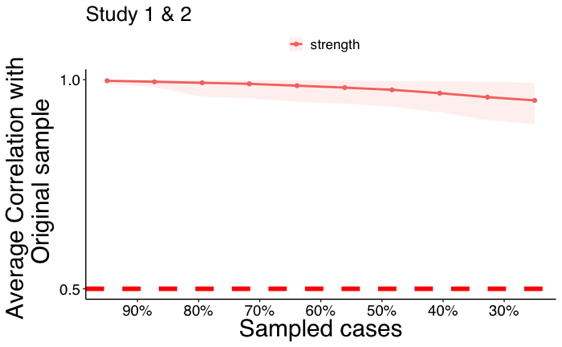


E F


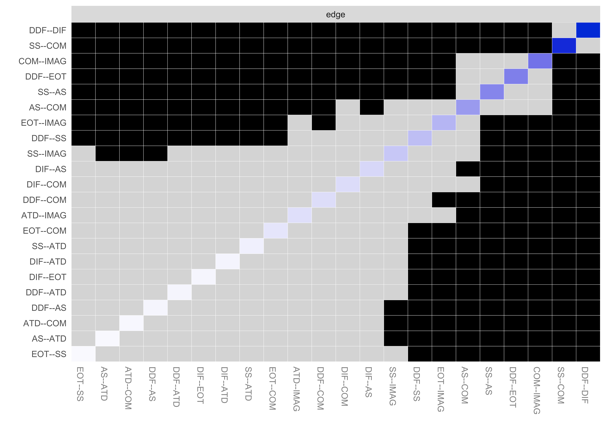

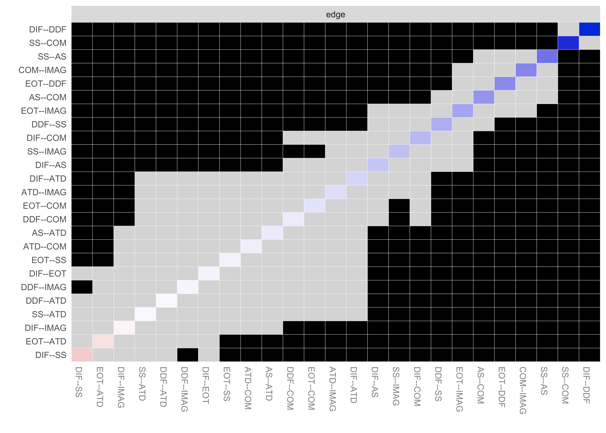


*Figure S.7.* *Network Stability.* A & B: 95% bootstrapped CIs around edge weights. C & D shows the correlation stability coefficient. All networks were estimated reliably. E & F: Edge difference plot, comparing edge pairs (strength of pairwise trait connections). Darker squares represent a significant (p <.05) difference between the pairwise edge comparisons. As in Study 1, trait connections within alexithymia and autism were significantly stronger than between alexithymia and autism connections.

*Network Comparison*

Study 1 & 2 networks were highly similar in structure, with a correlation of the edge weight matrixes of .88. The formal NCT showed no significant differences in network structure (M: 0.15, p = 0.05), but the network from Study 1 showed stronger average connectivity (3.48), than the network from Study 2 (2.82), S = .66, p = .01. There were no differences in individual edge invariance. Overall though, the separation of autism and alexithymia items and factors was replicated.

**CFA of Individual Measures**

*Autism Spectrum Quotient AQ-50*

We fitted 3 models based on the previous confirmatory studies on the AQ-50.

Model A.1, fitted the originally proposed 5-factor structure with no second-order terms (Baron-Cohen et al. 2001).

Model A.2. fitted an alternative model, with only a 3- factor solution (*social skills,* *attention* patterns and *communication*) suggested to outperform competing models and provide a more parsimonious measure (Russell 2012; English et al. 2020).

Model A.3, is a popular variation of the original AQ-50 factor structure specifying second order factors for all social related abilities, and a separate factor for attention patterns (Hoekstra et al. 2011).

Overall, Model A.2, with only a 3 factor solution and no second order factors was the best performing model. – Table S.7

*Table S.7.*

Model Comparison for AQ-50 CFA models

| Model | Df | AIC | BIC | Chisq | Chisq diff | Df diff | CFI | LTI | RMSEA | p |
| --- | --- | --- | --- | --- | --- | --- | --- | --- | --- | --- |
| Model A.2 | 296 | 85956.04 | 86243.32 | 2184.29 | NA | NA | .84 | .82 | .07[.06,.07] | ^***^ |
| Model A.1 | 1165 | 171541.75 | 172116.32 | 8402.08 | 6217.79 | 869^***^ | .63 | .61 | .07[.06,.07] | ^***^ |
| Model A.3 | 1170 | 171539.82 | 172088.26 | 8410.14 | 8.06 | 5^ns^ | .63 | .61 | .07[.06,.07’ | ^***^ |

*Note.* * < .05, ** < .01, *** < .001.

***Toronto Alexithymia Scale TAS-20***

We compared our models according to the original proposed structure for the TAS-20 (Bagby et al. 1994), measuring *difficulties identifying feelings* (DIF), *difficulties describing feelings* for the *externally-oriented thinking* EOT).

Model A.1. Included the 3 factors with no second-order factor

Model A.2. Specifying a higher order general factor as a latent cause for the specific factors.

Model A.3. Was a variation of A.1, with a method factor for the reversed scored items.

A bifactor model was also tested but it did not converge

Model A.1 was overall the best performing model. This model contained a simplified factor structure of only 3 AQ factors (Social, Attention patterns and Imagination) – Table S.8.

*Table S.8.*

Model Comparison for AQ-50 CFA models

|  | Df | AIC | BIC | X^2^ | X^2^ diff | Df diff | CFI | LTI | RMSEA |
| --- | --- | --- | --- | --- | --- | --- | --- | --- | --- |
| Model A.1 | 149 | 74153 | 74367 | 1193.8 |  |  | .88 | .86 | .07[.06,.08] ^***^ |
| Model A.2 | 152 | 75131 | 75329 | 2177.5 | 983.71^***^ | 3 | .76 | .73 | .10[.09,.10] ^***^ |
| Model A.3 | 150 | 77692 | 77954 | 1129.9 | -1047.56^ns^ | 8 | .89 | .87 | .07[.06,.07] ^***^ |

*Notes.* * < .05, ** < .01, *** < .001.
